# Spatial integration of multi-omics data from serial sections using the novel Multi-Omics Imaging Integration Toolset

**DOI:** 10.1093/gigascience/giaf035

**Published:** 2025-05-14

**Authors:** Maximilian Wess, Maria K Andersen, Elise Midtbust, Juan Carlos Cabellos Guillem, Trond Viset, Øystein Størkersen, Sebastian Krossa, Morten Beck Rye, May-Britt Tessem

**Affiliations:** Department of Circulation and Medical Imaging, NTNU–Norwegian University of Science and Technology, Trondheim, 7491, Norway; ELIXIR, Norway; Department of Circulation and Medical Imaging, NTNU–Norwegian University of Science and Technology, Trondheim, 7491, Norway; Clinic of Surgery, St. Olavs Hospital, Trondheim University Hospital, Trondheim, 7006, Norway; Department of Circulation and Medical Imaging, NTNU–Norwegian University of Science and Technology, Trondheim, 7491, Norway; Clinic of Surgery, St. Olavs Hospital, Trondheim University Hospital, Trondheim, 7006, Norway; Department of Circulation and Medical Imaging, NTNU–Norwegian University of Science and Technology, Trondheim, 7491, Norway; Department of Pathology, St. Olavs Hospital, Trondheim University Hospital, Trondheim, 7030, Norway; Department of Pathology, St. Olavs Hospital, Trondheim University Hospital, Trondheim, 7030, Norway; Department of Circulation and Medical Imaging, NTNU–Norwegian University of Science and Technology, Trondheim, 7491, Norway; Central staff, St. Olavs Hospital HF, Trondheim, 7006, Norway; ELIXIR, Norway; Clinic of Surgery, St. Olavs Hospital, Trondheim University Hospital, Trondheim, 7006, Norway; Department of Clinical and Molecular Medicine, NTNU–Norwegian University of Science and Technology, Trondheim, 7491, Norway; Clinic of Laboratory Medicine, St.Olavs Hospital, Trondheim University Hospital, Trondheim, 7006, Norway; BioCore–Bioinformatics Core Facility, NTNU–Norwegian University of Science and Technology, Trondheim, 7030, Norway; Department of Circulation and Medical Imaging, NTNU–Norwegian University of Science and Technology, Trondheim, 7491, Norway; Clinic of Surgery, St. Olavs Hospital, Trondheim University Hospital, Trondheim, 7006, Norway

**Keywords:** mass spectrometry imaging, image registration, spatial transcriptomics

## Abstract

**Background:**

Truly understanding the cancer biology of heterogeneous tumors in precision medicine requires capturing the complexities of multiple omics levels and the spatial heterogeneity of cancer tissue. Techniques like mass spectrometry imaging (MSI) and spatial transcriptomics (ST) achieve this by spatially detecting metabolites and RNA but are often applied to serial sections. To fully leverage the advantage of such multi-omics data, the individual measurements need to be integrated into 1 dataset.

**Results:**

We present the Multi-Omics Imaging Integration Toolset (MIIT), a Python framework for integrating spatially resolved multi-omics data. A key component of MIIT’s integration is the registration of serial sections for which we developed a nonrigid registration algorithm, GreedyFHist. We validated GreedyFHist on 244 images from fresh-frozen serial sections, achieving state-of-the-art performance. As a proof of concept, we used MIIT to integrate ST and MSI data from prostate tissue samples and assessed the correlation of a gene signature for citrate-spermine secretion derived from ST with metabolic measurements from MSI.

**Conclusion:**

MIIT is a highly accurate, customizable, open-source framework for integrating spatial omics technologies performed on different serial sections.

## Introduction

Detecting novel tissue biomarkers in cancer research is important for improving diagnosis and subsequent treatment choices. Spatial multi-omics has emerged as a promising methodological framework for identifying biomarkers within heterogeneous cancer tissue. Whereas the traditional use of single bulk omics technology, such as genomics, transcriptomics, metabolomics, or proteomics, could only capture one molecular level, multi-omics can elucidate complex biological processes occurring in the tumor tissue. Additionally, spatial omics technologies allow researchers to detect molecules within the spatial context of the tissue sample. In cancer research, spatially resolved methods are particularly powerful for analyzing the tumor microenvironment *in situ* containing a heterogeneous mixture of different cell types such as stroma cells, epithelial cells, and cancer cells of different aggressiveness. Spatial analyses are thus facilitating the identification of spatially defined molecular signatures, biomarkers, and microenvironmental cellular interactions that otherwise would be lost if using traditional bulk molecular analysis. Two prominent examples of such spatial omics techniques are 10x Genomics’ Visium Spatial Gene Expression assay [1] (ST) and mass spectrometry imaging (MSI) to detect spatial transcriptomics and spatial metabolomics data, respectively, together with tissue morphology. Even though this allows analyzing the link between single-spatial-omics and tissue morphology, to harness the full potential of such data and truly move toward spatial multi-omics, a reliable data integration methodology that accurately registers several spatial omics layers is required.

Collecting several different spatial omics data from the same tissue is often not possible due to incompatible sample preparation or destructive analyte extraction procedures, making it necessary to obtain serial sections, one for each omics layer, from the tissue of interest. Since standard histology staining can often be performed in the exact same section used for spatial omics analysis, stained images can be used to find a registration between serial sections, which are then applied to different spatial omics modalities. We refer to registration as the process of transforming one image to the same coordinate system as another. Another challenge is that different spatial omics platforms operate with different sampling resolutions and organizations. For instance, ST is organized as a sparse collection of equidistant spots of 55 µm diameter arranged in a hexagonal grid (ST-spots), and MSI consists of a dense map of pixels of 1–200 µm resolution (MSI-pixels). It is therefore necessary to establish a way of transforming between MSI-pixels and ST-spots after registration of stained images. ST and MSI have been integrated before, but these approaches have been performed semi-manually [2, 3] or relied on proprietary software [3, 4]. One alternative method performed ST and MSI on the same tissue section, removing the need for registration of serial sections [5]. However, this protocol limits the number of omics modalities that can be used. Therefore, we need novel highly accurate end-to-end pipelines that can handle the registration of serial sections, can include several omics methodologies, and can be automatic and openly accessible to the research community.

A significant challenge of registering serial sections is that the tissue composition between sections varies and potential deformation artifacts from sectioning can occur. This complicates an accurate registration between each neighboring section with increasing complexity as the distance between sections increases. Although several algorithms have been proposed to solve the task of registering serial sections [6–9], they are largely based on formalin-fixed, paraffin-embedded (FFPE) tissue sectioned at close distance (e.g., 4–6 µm). FFPE sections are far less fragile and prone to artifacts compared to frozen tissue required for many spatial omics measurements, therefore requiring robust registration methods.

**Figure 1:**
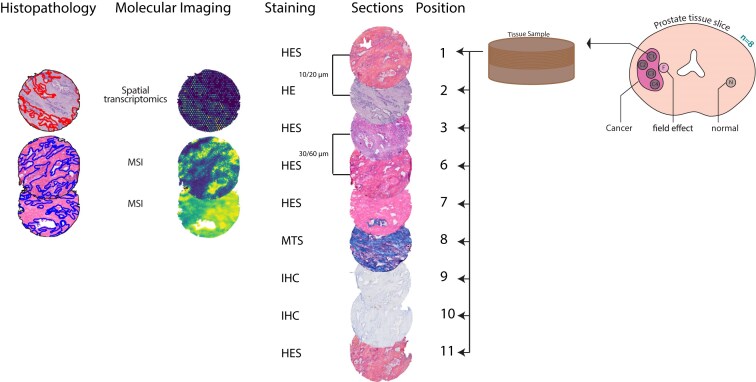
Overview of the ProstOmics spatial multi-omics dataset on prostate tissues. Sections of each core are numbered and processed in the same order. The sections used in this study are at positions 1–3 and 6–11 with a distance between 2 adjacent sections of 10–20 µm. The following staining techniques are used: hematoxylin, erythrosine, and saffron (HES); hematoxylin and eosin (HE); Mason’s trichrome staining (MTS); and immunohistochemistry (IHC). Spatial transcriptomics is applied to section 2, MSI in positive ion mode on section 6, and MSI in negative ion mode on section 7. Histopathology was evaluated for sections 2, 6, and 7.

Our group has generated a comprehensive and complex spatial multi-omics dataset as presented in Fig. 1 [10–12]. In this dataset, ST and MSI were performed on serial sections from cylindrical tissue samples that have an axial sectioning distance of up to 100 µm, which increases the difficulty of a viable registration due to stronger effects of tissue heterogeneity and tissue damage. In this study, we have tackled the challenges of spatial multi-omics data integration by developing 2 open-source applications. With our tool GreedyFHist, we were able to register stained heterogeneous serial sections that were up to 100 µm apart. GreedyFHist is compatible with most common image formats as well as ome.tiff and geojson [13], and it integrates well with QuPath [14], a popular open-source software for digital pathology. Building further on this framework, we developed the Multi-Omics Imaging Integration Toolset (MIIT), a framework capable of coordinating the different spatial resolutions and sampling organizations between different spatial modalities to create one spatial omics dataset. We used MIIT to define an end-to-end workflow for integrating ST and MSI by finding a robust registration between serial histology sections. Our integration works independently of the molecular properties measured, and new data types can be implemented to extend to other spatial omics technologies. Moreover, MIIT implements several utility functions for processing various spatial omics data types. To demonstrate the potential of MIIT, we investigated the correlation between a gene signature score for citrate and spermine secretion [15] from ST data with metabolic measurements from MSI in glands of cancer-free tissue samples from patients with prostate cancer. To calculate the gene signature scores, we used the single-sample gene set enrichment analysis (ssGSEA) [16].

## Results

Here we present a summary of GreedyFHist, our algorithm for registration of stained serial images, and MIIT, the framework for integration of spatial omics data. Detailed descriptions for both methods can be found in the methodology section.

### GreedyFHist for registration of histology images

**Figure 2:**
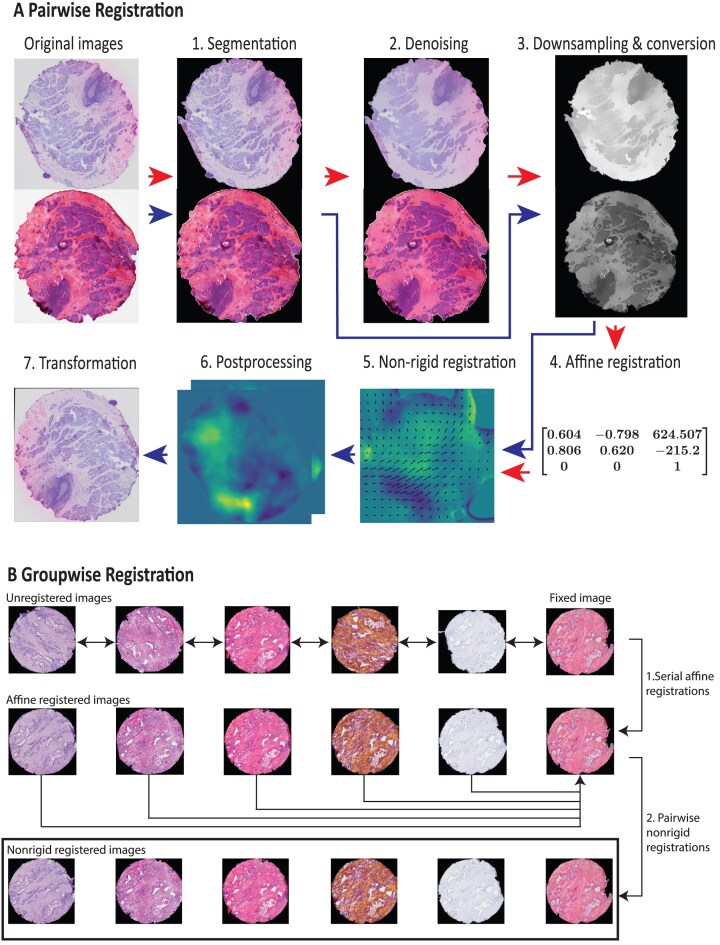
Overview of GreedyFHist’s registration. (A) Registration between 2 images. First, images are preprocessed for affine registration (red arrows). In step 1, images are segmented from background to focus on tissue region. Then, denoising (step 2) is applied to remove noise while retaining major histological features. Grayscale conversion and downscaling (step 3) are used on the images to improve registration time. Next, affine registration is performed using Greedy (step 4). Then images are preprocessed for nonrigid registration (step 1 and step 3; blue arrows). The affine transformation matrix and preprocessed images are passed to Greedy to compute nonrigid transformation matrices (step 5). Transformation matrices are then rescaled to the original image’s resolution, composited into one transformation matrix (step 6), and applied to the moving image (step 7). (B) Groupwise registration. When registering a series of stained images, we denote one image as the fixed image and every other image in the series as moving images. First, an affine registration between each neighboring image pair is computed. Then a transformation sequence is applied to each moving image to affinely register it to the fixed image (step 1). Finally, a nonrigid registration is performed between each affinely registered image and the fixed image (step 2).

GreedyFHist is our algorithm for registration of stained fresh-frozen serial images. Image registration is the process of finding a transformation from one image (*moving image*) to another (*fixed image*) such that the transformation applied to the moving image results in a warped image that is aligned with the fixed image. The main steps of the GreedyFHist algorithm are presented below and in Fig. 2:

1. **Segmentation:** Images are first segmented to remove any background noise and extract the tissue area using a segmentation algorithm based on the YOLO8 [17] model.
2. **Denoising:** Then images are denoised to remove unnecessary image features while keeping major histological morphology features intact.
3. **Grayscale conversion and downscaling:** In the last preprocessing step, images are converted to grayscale color space and downscaled to 1,024 × 1,024 pixel resolution to reduce the runtime of the registration.
4. **Affine registration:** During the affine registration, a global registration between the moving image and the fixed image is computed using the diffeomorphic registration tool Greedy [18].
5. **Nonrigid registration:** After the affine registration, preprocessing of images is repeated (steps 1 and 3) without denoising. Greedy is used to perform a nonrigid registration to align locally matching image features.
6. **Postprocessing:** The computed transformation matrices are rescaled to the moving and fixed image’s size and composited into one transformation matrix.
7. **Transformation:** Transformation matrices are used to transform images or pointset data from the moving image space to the fixed image space.

Furthermore, GreedyFHist includes a groupwise registration mode in which an ordered series of stained images is registered to a common fixed image. We denote the last image in the series as the fixed image:

1. **Serial affine registration:** On each pair of neighboring stained images, a pairwise affine registration is performed and consecutively applied on each stained image, resulting in an affine registration for the whole image series.
2. **Nonrigid registration:** Then, a nonrigid registration between each image in the series and the fixed image is performed. As with the pairwise registration, each transformation is composited to reduce interpolation errors.

GreedyFHist supports the most common image formats, including ome.tiff [19], and has support for applying registration to spatial data in image, pointset, and geojson [13] format by which it interfaces with image analysis software such as QuPath or ImageJ [20]. To further improve the runtime performance of the groupwise registration, we parallelized the serial affine and the nonrigid registration step using the multiprocess library [21, 22]. Because the task of registration for neighboring tissue sections can be applied to a variety of topics outside of the scope of this work, we provide GreedyFHist as a standalone application. Furthermore, GreedyFHist has the option to fine-tune regions of interest (ROI) in the histology images by supplying custom segmentation masks.

### MIIT

**Figure 3:**
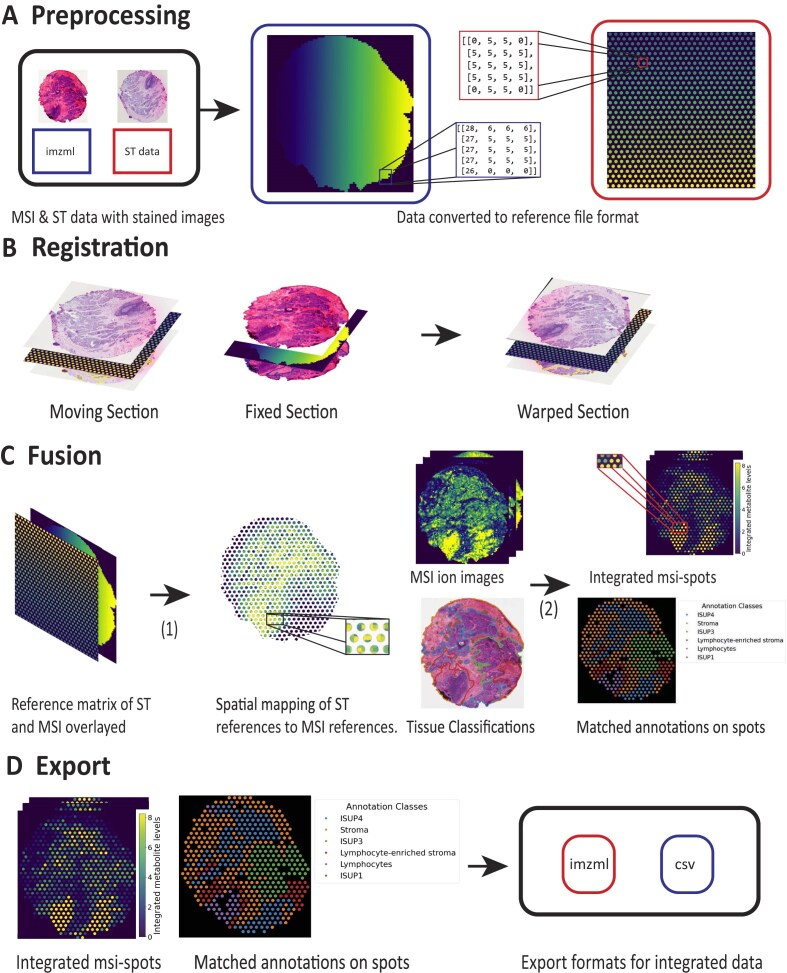
Integration workflow of MIIT. (A) Preprocessing. Preprocessing of ST and MSI data. File formats are processed to reference matrices and registered to stained images, if necessary. Each number in a reference matrix is either a reference to on-tissue molecular data or 0, which denotes background. In this example, ST contains 2,077 different spot references and MSI contains 7,527 different pixel references to molecular data points. Different references are highlighted in different colors. (B) Registration. Then the ST-section is registered to the MSI-section based on stained images. (C) Fusion. (1) Reference matrix of ST is used to group MSI-data within the same spot regions and (2) grouped MSI-data are aggregated within each spot, resulting in MSI-spots. If additional annotations are provided, integrated spots can be matched against these annotations as well. (D) Export. Lastly, integrated spots are exported into the relevant file formats.

MIIT is a framework for integrating spatial omics data from serial sections. An illustration of MIIT’s workflow for the integration of ST and MSI can be found in Fig. 3. By integrating, we refer to the registration of serial sections and accurate fusion of resolution and granularity between different types of spatial omics data. In the following, we use the term *section* to describe stained images, spatial omics data, and histopathology annotations that correspond to each other. We explain MIIT’s integration workflow by highlighting how a *source section* analyzed with MSI is integrated to a *target section* analyzed with ST:

1. **Preprocessing:** Spatial omics data are registered with stained images and a grid projection of the spatial omics data onto the image space of the stained image is performed, which allows accurate and efficient image transformations during registration.
2. **Registration:** Using the stained images as reference images, the source section is registered to the image space of the target section using GreedyFHist.
3. **Fusion:** Spatial omics data from the source section are fused to match the spatial organization of the target section’s spatial omics data and, optionally, additional histology annotations.
4. **Export:** Integrated spatial omics data from the source section are converted and exported to appropriate data formats for further analysis.

Although the main goal is to offer an efficient pipeline for the integration of spatial omics data, MIIT contains additional functions for processing spatial omics data and supports additional data formats such as pointset data, geojson, and tissue annotation masks that can be included in the integration process. Moreover, additional spatial data types and different types of spatial omics can be easily added by implementing interfaces that are provided in MIIT. Although we focus on the integration of ST and MSI, MIIT can also be used for implementing different integration workflows. We recommend using the modality with the highest spatial resolution as the source section and the modality with the lowest resolution as the target section.

### GreedyFHist registration outperforms alternative method

The registration of serial sections is the centerpiece of MIIT’s integration pipeline. Therefore, we evaluated GreedyFHist’s registration accuracy on our fresh-frozen prostate tissue samples. The evaluation of our tissue segmentation algorithm demonstrated a higher segmentation accuracy than an alternative based on Otsu’s thresholding [23] (Hausdorff distance: 69.159 µm [our method] vs. 140.814 µm [Otsu]; Supplementary Results).

**Figure 4:**
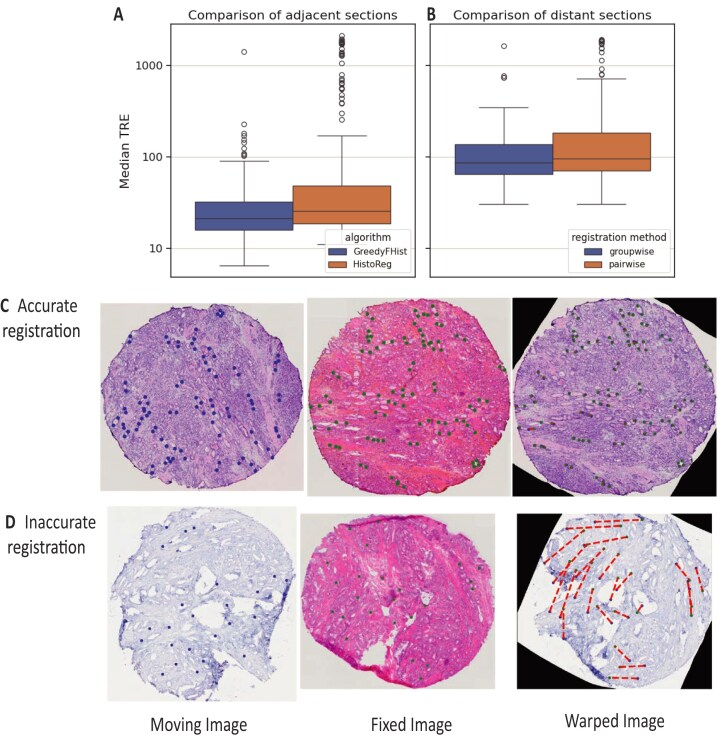
Assessing registration accuracy using landmarks. Comparison of error distribution in log_10_-scale for (A) registration of adjacent sections between GreedyFHist and HistoReg and (B) registration of distant sections between pairwise registration mode and groupwise registration mode using GreedyFHist. Median-TRE is shown at a log scale. Representative registration examples showing (C) an accurate registration (median TRE = 9.970 µm) and (D) an inaccurate registration due to tissue damage (median TRE = 593.006 µm). Landmarks of moving and warped landmarks are plotted in blue, landmarks of fixed images are green, and the distance between warped and fixed landmarks for warped images is illustrated in a red dashed line.

First, we investigated the registration accuracy of GreedyFHist for the registration on adjacent serial sections. For our test data, we selected each pair of adjacent sections (*n* = 7) from 32 samples (Fig. 1). If at least one of the images in each pair was too damaged for accurate registration, we discarded the pair. This resulted in 212 pairs of adjacent serial sections (distance of 10–20 µm) consisting of 4 different types of staining (Supplementary Table S1). We measured the accuracy for each registration by calculating the target registration error (TRE) between manually placed pairs of histologically distinct landmarks (Fig. 4C, D; average number of landmarks per section: 77). We compared GreedyFHist with HistoReg as both algorithms use the Greedy algorithm to compute the registration between preprocessed images and because HistoReg was the best open-source registration algorithm in the grand challenge for automatic nonrigid image registration (ANHIR) [7]. For each registration, we first calculated the median TRE of all landmark pairs. To evaluate the accuracy over the whole dataset, we then calculated the median of median-TRE (MM-TRE) and the average median-TRE (AM-TRE). GreedyFHist resulted in significantly lower median-TRE (MM-TRE: 21.025 µm vs. 25.529 µm, AM-TRE: 37.464 µm vs. 216.054 µm; *P* ≈ 1.045 × 10^−5^, 1-sided Wilcoxon signed-rank test; Fig. 4A, C, Supplementary Table S2) than HistoReg. GreedyFHist required on average 49.90 ± 6.74 seconds per registration compared to 57.16 ± 7.18 seconds with HistoReg. These results suggest that by applying our preprocessing method prior to image registration through Greedy, we can robustly register stained images of fresh-frozen serial sections with a faster runtime.

### Groupwise registration improves registration of distant neighboring section

In multi-omics experiments, it may be necessary to register stained images that are not adjacent neighbors but distant to each other. For instance, for the experimental setup depicted in Fig. 1, ST was applied on section 2 and MSI on sections 6 and 7 (which is equivalent to a distance of 40–100 µm). This motivated us to investigate how well distant sections can be registered with GreedyFHist. From 32 samples, we collected each image pair with a distance of 5 sections (*n* = 4) corresponding to 50–100 µm. Images that were too damaged for registration and images that GreedyFHist could only register poorly during the pairwise evaluation (median-TRE > 200 µm; Fig. 4D) were not included, resulting in 122 image pairs (Supplementary Table S3). We decided to analyze how well 2 distant images can be registered by evaluating 2 different strategies: *pairwise registration*, in which the moving image and the fixed image were registered directly, and *groupwise registration*, in which intermediate images were used to build a registration sequence between the moving and the fixed sections.

The groupwise registration resulted in significantly lower TRE-metrics than the pairwise registration (MM-TRE: 86.309 vs. 100.406; AM-TRE: 141.347 vs. 292.452; *P* ≈ 0.015 for median-TRE; Supplementary Table S4, Fig. 4B). Groupwise registration required on average 61.36 ± 4.12 seconds compared to 49.377 ± 4.970 seconds for pairwise registration. The higher TRE-metrics for the direct registration are expected due to the registration having to account for stronger effects of tissue heterogeneity, whereas the groupwise registration adjusts to the changing tissue heterogeneity stepwise with each intermediate registration. Moreover, the higher TRE-metrics between distant and adjacent tissue sections are also expected due to stronger effects of tissue heterogeneity in distant sections compared to adjacent sections. To conclude, this experiment shows that to overcome the issue of registration of distant images, a groupwise registration strategy yields significantly better results than aiming to register images directly.

### Spatial multi-omics and its integration using MIIT is capable of reproducing known biological associations

To demonstrate the applicability of MIIT to gain multi-omics biological insights, we chose to investigate a well-known metabolic mechanism of the prostate: citrate and spermine secretion. Prostate luminal cells secrete large amounts of the metabolite citrate into the prostate lumen. The high production of citrate is proposed to be the result of high zinc levels, which inhibits the citric acid cycle, causing accumulation of citrate. Spermine is another metabolite that is secreted at high levels by the prostate and is highly correlated with citrate levels [15, 24, 25]. Secretion of citrate and spermine is lost during progression to high-grade prostate cancer and is absent in the prostate stroma [26, 27]. Thus, high levels of citrate, zinc, and spermine are a hallmark of normal prostate glandular tissue [28, 29]. Our research group has previously developed a gene signature for citrate secretion gene signature (CSGS) in the prostate [15], which links gene expression to metabolic citrate secretion. To demonstrate the potential of MIIT, we investigated the correlations between ssGSEA scores [16] calculated from CSGS on ST data and citrate, zinc, and spermine levels from MSI [30].

For investigating the relation between CSGS and metabolite levels, we chose a subset of 7 cancer-free samples from 32 samples, where each sample was taken from different patients with prostate cancer. ST and MSI in positive and negative ion mode were integrated using MIIT as described in the Methods section with an average MM-TRE of 86.04 µm (range: 38.17–139.22 µm; Supplementary Tables S5, S6). As illustrated in Fig. 1, ST was performed on section 2 (ST-section), MSI in positive ion mode on section 6 (MSI-POS-section), and MSI in negative ion mode on section 7 (MSI-NEG-section). Spatial integration of these data resulted in 2 sets of MSI spots per sample, one for positive ion mode (MSI-POS-spots) and one for negative ion mode (MSI-NEG-spots). Citrate and zinc measurements were taken from integrated MSI-NEG-spots and spermine measurements from integrated MSI-POS-spots. Furthermore, histopathology annotations from ST-sections, MSI-POS-sections, and MSI-NEG-sections were used to classify each ST-spot, MSI-POS-spot, and MSI-NEG-spot as either gland or stroma. To account for batch effects that can occur in different samples, we analyzed each sample separately. Integrated spots that did not cover tissue regions or were not classified as gland or stroma were excluded from further data analysis. For computing ssGSEA scores for CSGS, we found 109 of 150 genes in CSGS in our ST data (Supplementary Table S7).

**Figure 5:**
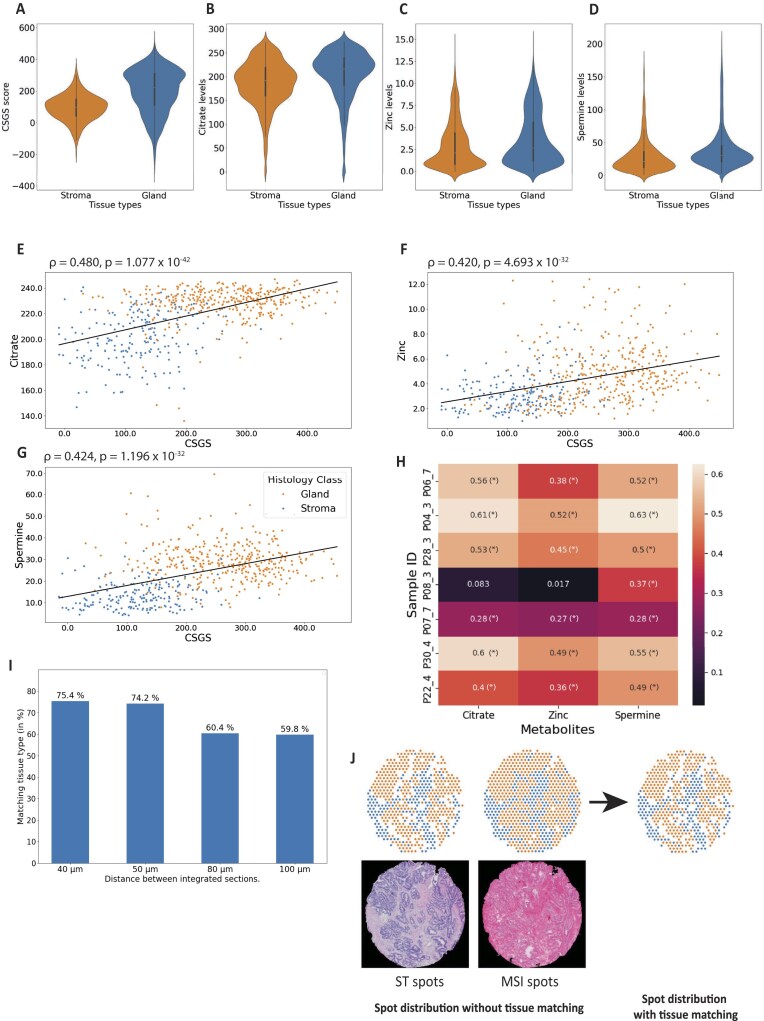
Comparing gene signature and metabolites between gland and stroma spots. Gene scores and metabolite levels in stroma and gland spots for (A) GSCS, (B) citrate, (C) zinc, and (D) spermine. (E) Citrate, (F) zinc, and (G) spermine levels plotted against CSGS score for one sample (P28_03) for integrated spots colored according to tissue type. Linear regression lines, Spearman correlation coefficient ${\mathrm{\rho }}$, and *P* value are shown. (H) Sample-wise correlation coefficients between CSGS and citrate, zinc, and spermine. (*) denotes significance (<0.05). (I) Percentage of successfully integrated spots after tissue type matching across different section distances. (J) Distribution of gland and stroma spots in ST-section and MSI-section without tissue type matching and after tissue type matching. Spots that could not be assigned to either stroma or gland or had a different histopathology classification were discarded beforehand.

Differential expression analysis of samples comparing gland and stroma spots predominantly showed significantly higher CSGS scores and higher citrate, zinc and spermine levels in glands (Mann–Whitney *U* rank test, Fig. 5A–D; Supplementary Fig. S1, Supplementary Table S8). Further, the CSGS score showed significant correlations with citrate, zinc, and spermine for most samples (Fig. 5E–H for 1 sample; Supplementary Figs S2, S3). This confirms that the well-known prostate mechanisms are present in this multi-omics dataset and that CSGS, citrate, zinc, and spermine are good candidates for a proof-of-concept demonstration of MIIT [26, 27].

### Tissue type matching improves correlation coefficients

**Figure 6:**
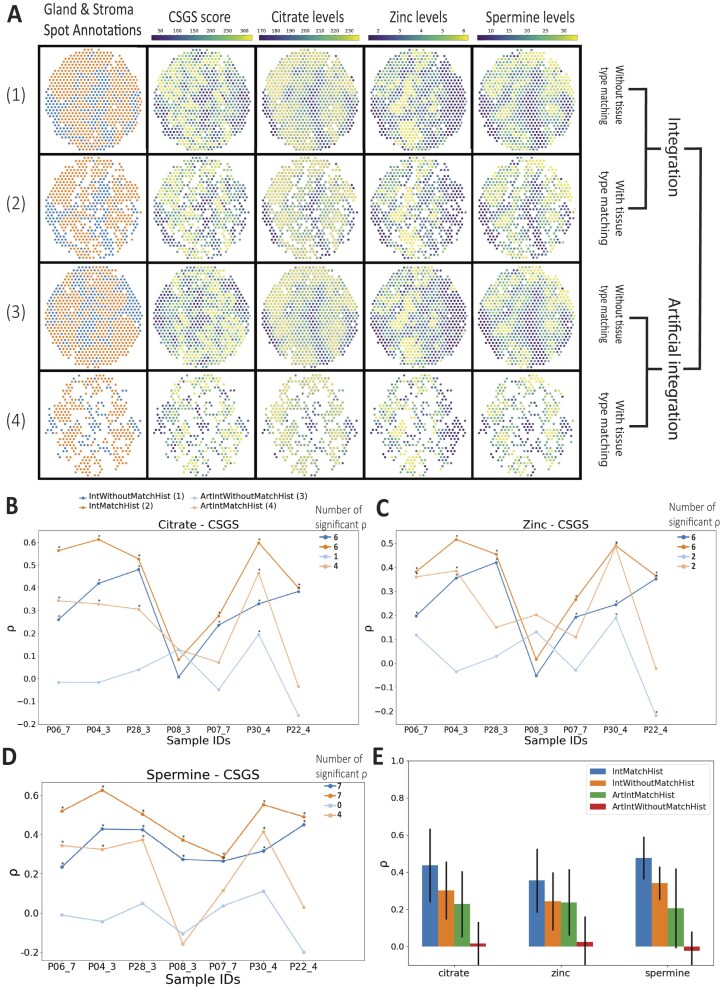
Comparison of different integrated spatial multi-omics datasets. (A) Spot-wise distribution of gland and stroma, CSGS, citrate, zinc, and spermine for 4 different datasets for sample P28_3. (B–D) Spearman correlation coefficient distribution for each sample for all 4 datasets. * denotes significant correlations (*P* < 0.001). (E) Average ${\mathrm{\rho }}$ for each metabolite compared to the CSGS score. Error bars represent standard deviation.

Despite a high integration accuracy, image registration is not able to account for all morphological changes in tissue composition between neighboring sections. For the 7 samples used in this analysis, the distance between the ST-sections and the MSI-sections ranged from 40 to 100 µm due to their sectioning order (Fig. 1) and varying number of discarded sections. This results in some areas where ST-spots and MSI-spots have a different tissue type (Fig. 5J). Therefore, we were interested in analyzing the effect of tissue heterogeneity between ST-section and both MSI-sections and whether the correlation analysis is impacted by this. From the initial integrated datasets (termed **IntWithoutMatchHist**), we removed all spots with nonmatching tissue types (termed **IntMatchHist**), which reduced the dataset by 33.8% for matching tissue types between ST-section and MSI-NEG-section and 32.96% between ST-section and MSI-POS-section (Fig. 5I). Spearman correlation analysis between the CSGS score and metabolite levels demonstrated that only including histology-matched spots increased correlations significantly for citrate ($\bar{\rho }$ = 0.437 vs. $\bar{\rho }$ = 0.302, *P* = 0.013, paired *t*-test on z-transformed ${\mathrm{\rho }}$), zinc ($\bar{\rho }$ = 0.356 vs. $\bar{\rho }$ = 0.244, *P* = 0.008), and spermine ($\bar{\rho }{\mathrm{\ }}$= 0.478 vs. $\bar{\rho }$ = 0.341, *P* = 0.006) (Fig. 6A–D (1,2), Supplementary Table S9, Supplementary Figs. S2, S3). These results show that taking changing histology into account when integrating serial sections is important when investigating tissue type–specific biological mechanisms.

### Correctly integrated data perform better than artificially integrated data, demonstrating proper integration is important

To test the robustness of MIIT, we compared the IntWithoutMatchHist and IntMatchHist with a dataset featuring deliberately poor integration. This artificially integrated dataset was created by rotating the ST-sections after registration by 180 degrees followed by integration, which is termed **ArtIntWithoutMatchHist**. Additionally, we performed tissue type matching on the artificially integrated data (**ArtIntMatchHist**) to investigate whether poorly integrated spots with the same tissue type give us similar results as correctly integrated spots. Not surprisingly, a much larger proportion of nonmatching spots had to be degraded when the data were artificially integrated (Fig. 6A, Supplementary Fig. S4, S5–S10). We evaluated all 4 datasets by comparing the mean Spearman correlation coefficient ${\mathrm{\rho }}$ and the number of significant correlations. For both criteria for all 3 metabolites, IntMatchHist performs best, followed by IntWithoutMatchHist, ArtIntMatchHist, and ArtIntWithoutMatchHist (Fig. 6B–E, Supplementary Table S10, Supplementary Fig. S11). Interestingly, both correctly integrated datasets showed better results than the artificial ones, showing that an accurate registration is necessary. The fact that IntWithoutMatchHist performed better than ArtIntMatchHist demonstrates that there is biological heterogeneity between spots of the same tissue type (gland and stroma areas). In other words, poorly integrating one gland spot with a different distant gland spot, for instance, can weaken the biological interpretation of the data.

## Discussion

In this article, we introduce MIIT, a novel and flexible framework designed to integrate various spatial omics data. Using MIIT, we defined a semi-automated workflow that merges ST and MSI data into a combined dataset. It is customizable and can be extended to other types of spatial omics data. Our proof-of-concept analysis demonstrated MIIT’s capability by integrating ST and MSI data to validate a bulk-generated gene signature for citrate and spermine prediction in prostate cancer [15, 25–29]. A key component of MIIT’s integration workflow is the registration of neighboring tissue sections using a novel nonrigid registration algorithm, GreedyFHist. We evaluated GreedyFHist on fresh-frozen tissue samples with 4 different types of staining, achieving a high accuracy. Both MIIT and GreedyFHist are available as open-source software.

Current algorithms tend to utilize computationally intensive deep learning methods for computing the registration [9], whereas we have opted for a lightweight algorithm that can run efficiently on a CPU architecture. GreedyFHist shares similarities with HistoReg, another state-of-the-art registration algorithm: both GreedyFHist and HistoReg utilize Greedy for computing affine and nonrigid registration parameters. However, by implementing a novel preprocessing pipeline, GreedyFHist outperforms HistoReg’s accuracy for pairwise registration on a set of fresh-frozen serial sections (MM-TRE: 20.988 µm vs. 25.369 µm; AM-TRE: 44.096 µm vs. 217.064 µm; Supplementary Table S2, Fig. 4A). GreedyFHist includes background segmentation to remove noise and center the registration on the tissue region of interest, followed by mean shift filtering to denoise features in tissue images while preserving major histological features. We further compute the center-of-mass to improve the initial alignment of the affine registration. These additional steps contribute to GreedyFHist’s higher accuracy compared to HistoReg. Based on the pairwise registration, we further developed a groupwise registration method that leverages intermediate stained images to accurately register stained images up to 100 µm apart. This makes it possible to accurately integrate spatial omics data distributed over several serial sections. GreedyFHist can process common formats in bioimaging analysis such as ome.tiff and geojson and apply transformations to various image and pointset data (e.g., simple coordinates and geojson). While several registration algorithms for histologically stained images have been proposed in recent years, only a few have made their code available [7, 9], offer built-in support for processing of bioimaging files types, and have the functionality to apply the computed transformation to various image (e.g., single- and multichannel images and annotations) and pointset data (e.g., coordinate data, geojson), which are necessary for spatial multi-omics integration [31, 32]. Through these features, GreedyFHist interfaces with the well-known bioimaging software such as QuPath and ImageJ and is available as a separate software package, where it can be used via command line and interactively via API. Therefore, it is also possible to use GreedyFHist outside of MIIT (e.g., for 3-dimensional image reconstruction).

For the fusion from the spatial organization of MSI to the spatial organization of ST data, we calculated weighted statistics between each ST spot and spatially matching MSI data. This approach is appropriate given the high-resolution dense representation of MSI data and the low-resolution sparse distribution of ST data. However, for use cases in which data are mapped from low to high resolution (e.g., ST into MSI), advanced methods such as deconvolution techniques could be included in the integration workflow. Future research could explore deconvolution methods that directly work on integrated molecular data.

Despite growing interest in spatial multi-omics analysis, few computational open-source end-to-end pipelines exist for integrating various types of spatial omics data. For instance, Sun et al. [3] performed spatial integration between ST and MSI data by manually registering MSI data onto a neighboring hematoxylin and eosin (HE)–stained image of the ST section. They used MassImager Pro for metabolomics and SCiLS Pro 2018b (Bruker Daltonics) for lipidomics. Unlike MIIT’s automated workflow, this is a manual process, making it challenging to apply to large-scale datasets and neglecting the changing tissue morphology between serial sections. Ravi et al. [2] integrate MSI with ST data by registration of neighboring stained serial sections to map MSI to ST data followed by averaging MSI pixels that share the same ST spot coordinates. However, they rely on an affine registration method [33], known to produce higher target registration errors than nonrigid registrations [6–8], and have not evaluated their method on distant sections. In contrast, MIIT employs GreedyFHist to achieve high accuracy through nonrigid registration and has successfully integrated ST and MSI at a distance of 100 µm. An end-to-end pipeline similar to MIIT is the SMOx pipeline, presented by Zhang et al. [4], which they used to integrate ST data and lipidomics via MSI in prostate cancer samples. They performed nonrigid registration of serial stained images to align MSI and ST data. For the fusion of MSI onto ST data, SMOx used granularity matching with a Gaussian approach, whereas MIIT used weighted statistics over the shared area between each ST spot and MSI pixels. However, the biggest difference between the 2 pipelines is that SMOx is proprietary, while MIIT’s framework is open-source and customizable, providing users with more flexibility. An alternative sample preparation protocol by Vicari et al. [5] enables the retrieval of matrix-assisted laser desorption/ionization time-of-flight (MALDI) MSI and ST from the same tissue section, circumventing the issue of registration of serial sections. Although this strategy limits the number of spatial omics types that can be used, it can be combined with MIIT by adding other types of spatial omics data to serial sections. Through MIIT’s customizable open-source design and the high registration accuracy provided by GreedyFHist, it is a viable alternative framework for spatial multi-omics integration.

Another excellent advantage of MIIT is its ability to extend seamlessly to other types of spatial omics with minimal prerequisites. This is achieved through grid projection into a standard reference matrix format, enabling spatial matching and nonrigid transformation of all spatial omics data with high accuracy. The registration between spatial omics data is performed by registering stained images, making the process independent of the omics methodology attached to each stained image. Molecular measurements are only accessed during the fusion step for mapping between different spatial omics types. The only prerequisite is that each spatial omics data requires a reference image for registration. Although we used stained histology images as references, other image types could also be used, presenting a topic for future research. The easiness of adding additional types of spatial omics makes MIIT a useful framework for exploring new types of spatial omics integration.

Nonetheless, GreedyFHist and MIIT have some limitations that we are addressing. Integrating multiple serial sections presents the challenge of unavoidable spatial alteration of histology. We found that including only integrated spots with consistent histology types between tissue sections significantly improved correlations between known associations of the citrate-spermine gene signature and the metabolites citrate, zinc, and spermine in prostate cancer tissue. However, this came at the cost of losing integrated spots. This loss was acceptable in our dataset due to the abundance of stroma and gland spots, but it could be problematic for smaller tissue components (e.g., perineural invasions on prostate tissue). Ensuring that molecular imaging experiments are performed on tissue sections as closely as possible is crucial to reduce the effects of tissue heterogeneity, a problem not unique to MIIT. At a certain distance in the z-plane, the biological differences between 2 sections become significant, making it more practical to analyze each section separately rather than integrating the data. The maximum distance at which 2 tissue sections can still provide valuable data integration with MIIT should be determined by the user, based on the specific tissue being analyzed and the research question at hand. Another limitation is the occurrence of tissue damage [7, 9]. Although GreedyFHist achieved high accuracy in registration, some images could not be registered accurately due to tissue damage (Fig. 4D), which can become problematic during groupwise registration. Developing methods to estimate registration quality and filter out unregistrable images could address this issue. These limitations affected MIIT directly since it required accurate registration for spatial multi-omics integration. MIIT addresses this issue through its flexible design, which allows us to exchange GreedyFHist’s registration with an alternative registration algorithm during registration. This alternative registration algorithm registers images using manually chosen landmarks (e.g., through external tools like Fiji [34]). MIIT has extra functionality to add custom registration algorithms easily to the integration pipeline, making it easily adaptable to specific use cases (e.g., for faster registration or different image modalities).

MIIT’s workflow is versatile, making no assumptions about the origin of the underlying data, making it valuable for a wide range of heterogeneous disease types. MIIT advances the investigation of the tumor microenvironment through spatial multi-omics integration of spatially resolved transcriptomics and metabolomics [35–37], enabling comprehensive computational analysis of different classes of molecules, and is useful for all types of tissues, not only cancer tissue. Future work will focus on analyzing the spatial relationship between molecules detected by different omics methods within the prostate tumor microenvironment to reveal insights into prostate cancer aggressiveness. Additionally, MIIT’s open and flexible design allows for future developments, such as adding automated tissue annotations [38] and integrating other types of molecular imaging data. MIIT could also be used to integrate spatial omics methods applied to whole organ tissue sections with magnetic resonance imaging (MRI) from the same organ, requiring algorithms capable of registering stained histology images with MRI data [39–41]. Establishing normalization methods for analyzing spatial multi-omics datasets is also necessary but outside this work’s scope.

To conclude, we presented a novel framework for the integration of spatially resolved molecular imaging. MIIT is openly accessible and customizable to handle a variety of different types of spatial omics data. We also developed GreedyFHist for the registration of tissue samples that achieved high accuracy on a set of fresh-frozen tissue samples and is available for all tissue types.

## Methods

### ProstOmics dataset

All data used in this study are part of the ProstOmics dataset, from our larger project “‘Tissue Is the Issue’: A Multi-Omics Approach to Improve Prostate Cancer Diagnosis” (ERC: 758,306). Parts of this project have been used in previous publications of our group [10, 42], but the setup for this study is presented in Fig. 1. Several parts of this dataset have been used in previous work of this group: HE- and Mason’s trichrome staining (MTS)–stained sections, histopathology on HE-stained sections, and ST data have been used by Andersen et al. [10]. ST data have been used by Kiviaho et al. [12]. ST (including HE staining) and MALDI-TOF MSI data in negative ion mode (including hematoxylin, erythrosine, and saffron [HES] staining) have been used by Krossa et al. [11].

### Patient inclusion and sample collection

All human prostate tissue material used in this study was collected after informed written consent was given by patients with prostate cancer undergoing radical prostatectomy. The regional ethics committee of Central Norway approved this research (identifier 2017/576). All methods were carried out according to national and EU ethical regulations. Human prostate tissue samples were collected after informed written consent was given by patients with prostate cancer undergoing radical prostatectomy. Samples of 8 patients, who were not treated prior to surgery, were collected from St. Olav’s hospital, Trondheim, Norway, between 2008 and 2016. Three patients were considered relapse-free as no confirmed relapse occurred after 12 years and 5 patients were classified as relapse as metastasis had occurred within 3 years after surgery. A 2-mm-thick slice was cut from the middle of the prostate (transverse plane), snap frozen, and stored at −80°C as described by Bertilsson et al. [43] immediately after surgery by expert personnel at Biobank1, St. Olav’s University Hospital, Trondheim, Norway. A range of 8–13 tissue samples (3 mm in diameter) were collected from each fresh-frozen tissue slice, using an in-house built drill system. Based on HES-stained tissue annotations, 4 sample cores were selected from each patient’s tissue slice with 2 samples containing cancer tissue, 1 sample being cancer tissue adjacent and 1 sample from a faraway region on the tissue slice containing no cancer tissue, giving a total of 32 samples.

### Cryosectioning

Tissue sections from each tissue sample were cut with a 10-µm thickness inside a cryostat at −20°C (Cryostar NX79; Thermo Fisher Scientific). For this study, we selected sections (positions 1–3 and 6–11) from various staining methods presented in Fig. 1. In total, we used 288 sections from all 8 patients where 9 sections were collected from each tissue core. Four conductive slides were vacuum packed and stored at −80°C until further use.

### Staining methods

All sections were stained for various experiments with HE (*n* = 32), HES (*n* = 160), immunohistochemistry (IHC staining for lipopolysaccharides (LPS) and lipoteichoic acid (LTA); *n* = 61; Supplementary Methods), and MTS (*n* = 32) and scanned at 20× magnification using a High Throughput Slide Scanner VS200 (Olympus). We note that sections 3, 6, and 7 were stained after performing MALDI-TOF MSI (*n* = 68). Additionally, HES-stained tissue of section 3 (*n* = 32) was heated for 5 minutes at 95°C before MALDI-TOF MSI according to the protocol developed by Høiem et al. [42]. MALDI-TOF MSI from section 3 was not used in this study. For each core, sections were stained in the same order (see Fig. 1).

### Histopathology

HE scans from section 2 and HES scans from sections 6 and 7 were independently evaluated and annotated by 2 experienced uropathologists using QuPath version 0.2.3 and 0.4.4. The identified areas were lymphocytes, stroma, epithelial areas, glands, and tissue borders in addition to cancer areas according to the Gleason Grade Group system. Furthermore, a consensus pathology evaluation was reached in agreement with both pathologists. For each ST spot from section 2, the fraction of different tissue types and regions present was calculated.

### Spatial transcriptomics

Sequencing libraries were created from the tissue sections by using the Visium Spatial Gene Expression Slide & Reagent kit (10x Genomics, product code: 1,000,184) following the manufacturer’s manual. In brief, tissue sections were fixed using methanol, followed by HE staining, and scanning of slides at 20× magnification. For microscopic scanning, a coverslip was put over the sections and removed afterward. To capture mRNA, tissue sections were incubated with permeabilization enzyme for 12 minutes, which previously had been optimized using the Visium Spatial Tissue Optimization Slide & Reagent kit (10x Genomics, product code 1,000,193). A second strand mix was added to create a second strand, followed by amplification of cDNA by real-time quantitative PCR (qPCR). The amplified cDNA library was quantified with qPCR using the QuantStudio 5 Real-Time PCR System (Thermo Fisher), and the cDNA libraries were stored at −20°C until further use. Paired-end sequencing was performed on an Illumina NextSeq 500 instrument (SY-415–1001; Illumina) using the NextSeq 500/550 High Output kit v2.5 (150 cycles; product code 20,024,907). Each distinct coverage area (ST-spot) had a radius of 55 µm, and the distance between the center of each ST-spot was 100 µm.

### MALDI-TOF MSI

For MALDI-TOF MSI, 2 sections from each core were used, with position 6 for positive ion mode and position 7 for negative ion mode, resulting in a total of 64 sections. Before matrix application, all vacuum-packed slides with tissue sections were left on the benchtop for at least 20 minutes before opening the vacuum bag. Two different matrices, 2,5-dihydroxybenzoic acid (DHB) and N-(1-naphthyl) ethylenediamine dihydrochloride (NEDC), were prepared by dissolving DHB in 70% methanol/0.1% trifluoroacetic acid (concentration 20 mg/mL) and NEDC in 70% methanol (7 mg/mL). The HTX TM-Sprayer system was used to spray the matrix onto the tissue sections, with 14 and 18 layers of matrix for DHB and NEDC, respectively. The rapifleX MALDI Tissuetyper (Bruker Daltonics) equipped with a 10-kHz laser was used to measure all tissue sections, shooting 200 shots per pixel at a 10-kHz frequency with a spatial resolution of 30 μm. Prior to all measurements, the instrument was calibrated using red phosphorus. Tissue sections covered with DHB matrix were measured in positive ion mode with a mass range of *m/z* 100–1,000, while tissue sections covered with NEDC matrix were measured in negative ion mode with a mass range of *m/z* 40–1,000. All measurements included separate matrix-only regions. After data acquisition, the slides were stored at 4°C until staining with HES. The MSI data were first imported into FlexImaging (Version 5.0; Bruker Daltonics), where we applied binning (factor 0.8). Then, MSI data were imported into SCILS Lab (Version 2024a Pro), baseline corrected using top-hat, normalized using root mean square, and quantified intensities as max peak height. We selected masses that we previously identified on a similar fresh-frozen prostate tissue dataset where on-tissue tandem mass spectrometry and accurate mass acquisition with a high mass resolution instrument (MALDI-Orbitrap) were used to confirm analyte identity (Supplementary Fig. S12) [44]. Zinc was detected in negative ion mode in the form of ZnCl_3_^−^ and was identified through accurate mass and the isotopic pattern as described by Andersen et al. [45]. The mass identification in our previous studies was performed on prostate tissue sections retrieved and stored the same way and covered with the same matrices, NEDC and DHB for negative and positive mode, respectively. MSI data were exported in imzML [46] along with tissue border annotations in SCILS’s srd format. For this study, we extracted the peak intensity of citrate (*m/z* 191.0 [M-H]^−^) and zinc in the form of ZnCl_3_^−^ (*m/z* 174.8 [M]^−^) from the negative ion mode data and spermine (*m/z* 203.2 [M+H]^+^) from the positive ion mode data.

### Preprocessing of stained histology images and generation of tissue masks for segmentation training

Tissue masks that separate tissue and nontissue area (i.e., background) were used as training data for training our YOLO8-based segmentation model. They were created in QuPath (v 0.4.4). To speed up the process of annotating tissue area in histology images, we used a pixel thresholder to generate initial masks, which were then manually corrected for misclassified areas. The pixel thresholder “Create Objects” function (options: minimum object size: 40,000 µm^2^; minimum whole size: 1000 µm^2^) was used on the average intensity value of each pixel after applying a Gaussian filter (sigma: 1). The thresholds for HE, HES, and MTS were set between 208 and 215; for IHC, the threshold was set between 218 and 225 due to lower staining intensity. The threshold was manually adjusted on each tissue image if necessary. Misclassified areas (e.g., due to noise in image or low staining intensity) were manually corrected using QuPath’s annotation tools. Stained images, tissue masks, and histopathology were exported using a groovy script [47] to *tiff* images. The resolution for each image was set to 1 pixel per µm.

### Hardware setup

All experiments were performed using the same hardware: Intel Ice Lake Xeon Processor (32-core; 32 threads; 2.1 GHz).

### Pairwise tissue registration—GreedyFHist

#### Tissue segmentation

To remove background noise from the image and center the image around the tissue area, we performed background segmentation: images were resampled to a resolution of 640 × 640 and converted to grayscale. Spurious noise from the background of the image was removed using a method by Chambolle [48] that performs total variation denoising [49]. Then we used the YOLO8 model to perform tissue segmentation on the image. The model was trained for tissue segmentation on our dataset. From the segmented image, small classification artifacts (e.g., due to contamination on the slide) were filtered out by removing any region with a smaller area than 10,000 µm^2^. The area threshold is dependent on the input data and can be adjusted by the user. The tissue mask was then resampled back to its original size. Since the process of identifying correct tissue masks can vary between different experiments, we have implemented the option for users to supply their own tissue mask (e.g., from manual annotations) in GreedyFHist. This flexibility allows the user to apply masks for selected region of interest (ROI) on the tissue. After applying tissue masks to crop images, both images were padded to the same uniform shape.

#### Denoising

Stained images are usually high-resolution images containing a large amount of detail. During registration, such details may act as noise, which negatively influences registration accuracy. Therefore, removing such noise while keeping major histological features intact is required. In the next step, we applied OpenCV’s implementation of mean shift filtering [50] as a method of denoising: (i) Images are resampled to 512 × 512 pixels to improve filtering speed. (ii) Images are converted to the HSV color space [51]. (iii) Mean shift filtering requires a spatial window radius and color window radius that determine the degree of filtering. We set these parameters for the spatial window radius to 20 and for the color window radius to 15. (iv) Images are converted back to RGB color space and resampled to their original size.

#### Grayscale conversion and downsampling

After denoising, images are smoothed with a Gaussian kernel (sigma: 2) to avoid aliasing effects during downsampling, resampled to a resolution of 1,024 × 1,024 pixels, and converted to grayscale to reduce image complexity further. Images are then padded with 100 pixels on all sides to leave room for deformations during registration.

#### Affine registration

We performed an affine registration to find a global alignment between the moving image and the fixed image. For computing the registration between 2 preprocessed images, we use Greedy [52], a registration tool that implements the greedy diffeomorphic registration algorithm. Greedy was originally developed for medical image registration but was also used for the registration of stained images in a previous study [6]. We adopt some findings from this study, namely the use of the NCC kernel metric as a similarity measure and the postprocessing to rescale the computed transformation matrices to the image’s original resolution. We use Greedy in version 1.0.1, although other versions should be compatible with our registration pipeline as well.

Preprocessed images are exported in the nifty format using SimpleITK [53–55]. We initiated Greedy with a transformation that centers both images at their center-of-mass, which we computed using the tissue masks. Then Greedy performs a random search to find a suitable rigid registration, followed by a multiresolution pyramidic approach for the affine registration: the registration is initially computed on downscaled images, which is then recursively refined on upscaled images until the full resolution is reached. Greedy computes the registration using the limited-memory Broyden–Fletcher–Goldfarb–Shanno algorithm [56, 57], and we used the NCC kernel metric with a kernel size of 1% of the preprocessed image resolution (i.e., 10 pixels). Overall, we achieved the best result with the scaling factors 8, 4, 2, and 1, although in some instances, scaling factors 4, 2, and 1 resulted in more accurate registrations.

#### Nonrigid registration

After the affine registration, a nonrigid registration is performed to find a nonuniform transformation that can align the moving and fixed image locally, which improves on the previous global registration [58]. For this, input images are preprocessed again by repeating step 1 (tissue segmentation) and step 3 (downscaling and grayscale conversion) without denoising. Due to the locality of the nonrigid registration, including all image features results in more accurate transformation matrices. The same parameters as for the affine registration were applied for the nonrigid registration. We additionally initialized the registration by supplying the computed affine registration and set 2 regularization parameters (pre-sigma: 5, post-sigma: 4). Supplementary Table S11 contains all parameters passed to Greedy for the affine and nonrigid registration.

#### Postprocessing after affine and nonrigid registration

Tissue registration will result in 2 transformation matrices, 1 for affine and 1 for nonrigid registration. We rescaled both transformation matrices to match the original image’s sizes. The cropping and padding operations applied during preprocessing are expressed as translation transformations using SimpleITK [54, 55]. These transformation matrices are composited along with the affine and nonrigid transformation matrices into a single displacement field matrix. This reduces interpolation artifacts during transformation as well as the number of image operations applied during transformation from moving to fixed image space.

#### Transformation from moving to fixed image space

Using SimpleITK, the displacement field is applied to warp image data from the moving to the fixed image space. GreedyFHist supports image data, image masks with annotations, pointset data in tabular form, and pointset data in the geojson format. For image and pointset data, linear interpolation is used, and for annotation masks, nearest-neighbor interpolation is used.

### Registration evaluation measures

The registration performance of GreedyFHist was evaluated using the target registration error (TRE), a common method for evaluating image registration algorithms such as in the ANHIR challenge [7, 8]. Three annotators placed an average of 77 landmarks between each pair of serial sections using the BigWarp plugin in Fiji (version 2.14.0/1.54f). Histological landmarks were chosen based on visually distinct histological features that are present in adjacent tissue sections, such as distinct glandular shapes, gland and stroma formations, and high-density clusters of cells, but also more nuanced image features, such as perforations in glandular structures. This ensured that both prominent and subtle image features were evaluated for registration performance. We aimed to annotate the same landmarks throughout serial sections and distribute them over the whole tissue area. If a landmark could not be located in an adjacent section (e.g., due to the disappearance of a gland, the tissue being too homogeneous to identify the distinct feature, or disappearance due to tissue damage), a new landmark pair was set when possible to keep the total number of landmarks consistent. After the initial placement of landmarks on serial sections, landmarks were cross-validated by visual inspection by a different annotator who had not worked on this set of serial sections. Although annotators mostly agreed on the placement of landmarks, a consensus was reached whenever annotators disagreed. The TRE is defined as


\begin{eqnarray*}
TRE_l^{wf} = {\left| {\left| {x_l^w - x_l^f} \right|} \right|}_2
\end{eqnarray*}

where $x_l^w$ and $x_l^f$ are a pair of matching landmarks *l* from warped and fixed landmarks. $|| . |{ |}_2$ denotes the Euclidean distance. We evaluated each registration pair using the median TRE,


\begin{eqnarray*}
\textit{median}\ TR{E}^{wf} = \mathop {{\mathrm{median}}}\limits_{l \in \mathcal{L}} TRE_l^{w,f}\ ,
\end{eqnarray*}

where $\mathcal{L}$ is the set of all landmarks between a pair of fixed and warped images. To evaluate the accuracy over the whole dataset, we calculate the median of median-TRE,


\begin{eqnarray*}
MM - TRE = \mathop {{\mathrm{median}}}\limits_{\left( {w,f} \right) \in \ \mathcal{P}} \left( {\textit{median}\ TR{E}^{w,f}} \right)\ ,
\end{eqnarray*}

and the average median-TRE,


\begin{eqnarray*}
AM - TRE = \mathop {{\mathrm{mean}}}\limits_{\left( {w,f} \right) \in \ \mathcal{P}} \left( {\textit{median}\ TR{E}^{w,f}} \right)\ ,
\end{eqnarray*}

where $\mathcal{P}$ is the set of all registration pairs. Since all images are exported at a resolution of 1 µm per pixel, all errors are computed in µm.

### MIIT—Multi-Omics Imaging Integration Toolset

#### Preprocessing

The main purpose of this step is to set up easily generalizable data formats necessary for integration and to ensure a spatial alignment between stained and molecular imaging data.

##### Reference file format conversion

Spatial omics data are often stored in parametric data formats (e.g., ST-spots are circles with a center and a diameter, whereas MSI-pixels are described by a pixel location and resolution such that 1 pixel occupies 1 MSI spectrum). To allow for accurate nonrigid transformations, we performed a grid projection in which spatial omics data are translated into a reference matrix format. In this format, the coverage of each data point in the image space of the stained histology is computed. We initialize an empty matrix (*reference matrix*) with the same resolution as the stained histology. Then, for each pixel that matches the coverage of a spatial data point, we place a reference to the matching spatial data point. This ensures that spatial omics data can be transformed accurately during the nonrigid registration and allows fine-grained fusion between spatial omics data points. In this format, each spatial omics data point is represented by a reference and projected onto a matrix with the same resolution as the stained image.

##### Registration between stained image and molecular data

It is necessary to ensure that stained image and spatial omics data are spatially aligned before integrating can proceed. ST is preprocessed using 10x Genomics Space Ranger (Version 1.0.0) [59], which also guarantees an accurate registration between stained image and ST-spots. For the registration of MSI data to the stained image, MIIT uses a registration method that is based on Verbeeck et al. [60, 61] (Supplementary Fig. S13):

1. The HES-stained image is segmented using the YOLO8-based tissue segmentation method described in the preprocessing section of GreedyFHist and then cropped to the tissue’s boundary section.
2. A feature image for the MSI data is derived from the first principal component of the MSI data’s PCA spectrum. The feature image is rescaled to the stained image’s resolution (e.g., 1 µm per pixel).
3. Both images are padded symmetrically to create a uniform shape and then padded with 100 pixels to allow room for deformations. NiftyReg [62] is used to find a rigid registration with the stained image used as the fixed image and the feature image as the moving image. We opted for a rigid registration, which only performs translation and rotation for image registration. This reduces deformation effects to the MSI-pixels, although options for affine and nonrigid registration can be selected as well.

Alternatively, if segmentation masks for the stained image or MSI data are available, these can be used instead for registration.

#### Registration

The goal of this step is to register the source section with the target section. This is achieved by using GreedyFHist to register the stained images of the *source section* and the *target section*. The resulting transformation is then applied to all spatial data types in the source section.

In the case that GreedyFHist’s registration fails (e.g., due to tissue damage), we also provide an alternative registration algorithm based on scikit-image and manually provided landmarks (e.g., from Fiji [34]) (Supplementary Methods).

#### Fusion

In this step, the spatially aligned data are used to transform the spatial organization of the spatial omics data from the source section to the target section. For each data point in the target data space, the overlap with data points in the source data space is computed. We chose to use the modality with the lowest resolution (ST, 55-µm circular spots placed in a hexagon pattern 100 µm apart) as the target section and the modality with the finest resolution (MSI, 30-µm square pixels with no space between) as the source section. Each individual overlapping area in the source data space is then aggregated based on basic statistical descriptors. In practice, this means that the overlapping area between each ST-spot and the registered MSI-pixels is computed. Due to performing the grid projection in step 1, we can use the overlapping area between the reference matrices to calculate the fraction that each MSI-pixel contributes to the shared area with each ST-spot. Then, we merge each metabolite intensity from the MSI space to the ST space by computing weighted statistical features (i.e., minimum, maximum, mean, standard deviation, median) over the shared area using the area fraction of each MSI-pixel as weight. We refer to the integrated MSI data as *MSI-spots*.

#### Export

For the integration of ST spatial transcriptomics and MSI, we export integrated data into a table format that uses barcodes from the ST count matrices as identifiers. Other export options exist as well: data can also be exported as an image stack with dimensionality n × w × h, where w and h refer to the dimensionality of the target section image space and n refers to the size of each data point (e.g., the number of spectra in each MSI-spot). MSI integrated data can also be exported in the imzML format.

### Analysis of the integrated spatial multi-omics dataset

After spatial integration, we applied tissue masks to all spots and filtered out any spots with less than 80% tissue coverage. Two samples (P08_3, P22_4) contained spots that were classified as “lymphocytes.” These spots were removed from the analysis. Correlations measured between CSGS and citrate, zinc, and spermine were computed using Spearman correlation.

## Availability of Source Code and Requirements

### MIIT

Code, tutorials, and instructions for setting up docker images with examples are available on GitHub. An archival copy is available via SoftwareHeritage [63].

- Project name: MIIT
- Project homepage: https://github.com/mwess/miit
- RRID:SCR_026476
- bio.tools: miit
- Operating system(s): Linux (if installed natively), Platform independent (if docker image is used)
- Programming Languages: Python 3.10
- Other requirements: GreedyFHist 0.0.3
- License: MIT

### GreedyFHist

Code, tutorials, and instructions for setting up docker images with examples are available on GitHub. An archival copy is available via SoftwareHeritage [64]. DOME-ML (Data, Optimization, Model and Evaluation in Machine Learning) annotations are available via the DOME registry under accession vsx18unv9t [65].

- Project name: GreedyFHist
- Project homepage: https://github.com/mwess/GreedyFHist
- RRID:SCR_026477
- bio.tools: greedyfhist
- Operating system(s): Linux (if installed natively), Platform independent (if docker image is used)
- Programming Languages: Python 3.10
- Other requirements: Greedy 1.0.1
- License: MIT

## Supplementary Material

giaf035_Supplemental_Files

giaf035_Authors_Response_To_Reviewer_Comments_Original_Submission

giaf035_Authors_Response_To_Reviewer_Comments_Revision_1

giaf035_GIGA-D-24-00380_Original_Submission

giaf035_GIGA-D-24-00380_Revision_1

giaf035_GIGA-D-24-00380_Revision_2

giaf035_Reviewer_1_Original_Submission Hua Zhang -- 11/20/2024

giaf035_Reviewer_1_Revision_1 Hua Zhang -- 2/24/2025

giaf035_Reviewer_2_Original_Submission Santhoshi Krishnan -- 11/26/2024

giaf035_Reviewer_2_Revision_1 Santhoshi Krishnan -- 2/25/2025

## Data Availability

A reduced and anonymized exemplary test dataset for exploring the use and functionality of MIIT can be found on Zenodo [66]. The full data utilized and analyzed in this study include sensitive information, and their management must comply with the General Data Protection Regulation (GDPR), Norwegian law, and specific patient consent and ethical approval. Consequently, the data are legally subjected to restricted access. Raw and processed transcriptomics data have been deposited at Federated European Genome-Phenome Archive (FEGA) Norway and are findable on the EGA portal (ega-archive.org) under the study ID EGAS50000000413. The spatial transcriptomics is deposited with the accession number EGAD50000000603. Data access can be requested through the EGA portal, where any data request will be processed through a data access committee at NTNU. Mass spectrometry data, stained images, and tissue annotations are not externally archived as there is currently no suitable public data repository that allows storage of these data that meets the data-sharing criteria postulated by the study’s ethical approval, patient consent, GDPR, and Norwegian law. Mass spectrometry data, stained images, and tissue annotations can be requested via email to maria.k.andersen@ntnu.no and may-britt.tessem@ntnu.no. For both archived and nonarchived data, access will only be granted after the following steps have been achieved: (i) The data requester and the intended use of the data must comply with GDPR regulation, Norwegian law, and the specific patient consent; (ii) data sharing with the specific data requester must be approved by the regional ethical committee (REC) in Norway; (iii) the Data Protection Impact Assessment (DPIA) may require revision; and (iv) there must be a signed data transfer agreement between the institution of the data requester and NTNU. Depending on the intended use of the data, the data requester can also be required to establish a collaboration agreement with NTNU before data sharing.

## Additional Files

**Supplementary Fig. S1**. Sample-wise gene scores and metabolite levels in stroma and gland spots for (a) GSCS, (b) citrate, (c) zinc, and (d) spermine. (*) indicates significance according to the Mann-Whitney U rank test (P < 0.05).

**Supplementary Fig. S2**. Citrate (a, d, g), zinc (b, e, h), and spermine(c, f, i) levels plotted against CSGS score for samples P06_07 (a, b, c), P04_3 (d, e, f), and P08_3 (g, h, i) for integrated spots colored according to tissue type. Linear regression lines are shown.

**Supplementary Fig. S3**. Citrate (a, d, g), zinc (b, e, h), and spermine(c, f, i) levels plotted against CSGS score for samples P07_07 (a, b, c), P30_4 (d, e, f), and P22_4 (g, h, i) for integrated spots colored according to tissue type. Linear regression lines are shown.

**Supplementary Fig. S4**. Number of spots classified as glands or stroma with and without tissue type matching as well as after normal and artificial integration.

**Supplementary Fig. S5**. Spot-wise distribution of gland and stroma, CSGS, citrate, zinc, and spermine for 4 different datasets (sample P06_7).

**Supplementary Fig. S6**. Spot-wise distribution of gland and stroma, CSGS, citrate, zinc, and spermine for 4 different datasets (sample P04_3).

**Supplementary Fig. S7**. Spot-wise distribution of gland and stroma, CSGS, citrate, zinc, and spermine for 4 different datasets (sample P08_3).

**Supplementary Fig. S8**. Spot-wise distribution of gland and stroma, CSGS, citrate, zinc, and spermine for 4 different datasets (sample P07_7).

**Supplementary Fig. S9**. Spot-wise distribution of gland and stroma, CSGS, citrate, zinc, and spermine for 4 different datasets (sample P30_4).

**Supplementary Fig. S10**. Spot-wise distribution of gland and stroma, CSGS, citrate, zinc, and spermine for 4 different datasets (sample P22_4).

**Supplementary Fig. S11**. (a–c) *P* values for testing whether the difference of Spearman correlation between 2 datasets is significant.

**Supplementary Fig. S12**. Tandem mass spectrometry for metabolite identification. Mean on-tissue MALDI MS/MS spectrum of (a) *m/z* 191.02 in negative ion mode identified as citrate [M-H]^−^ and (b) *m/z* 203.2 in positive ion mode identified as spermine [M+H]^+^.

**Supplementary Fig. S13**. Stained image is segmented from background region. For the imzml file, a feature image is derived based on the PCA decomposition. Then both images are padded symmetrically to a uniform shape and registered using NiftyReg. The resulting transformation is applied on the imzml data.

**Supplementary Fig. S14**. Validation of bounding box segmentation of stained image. Blue highlights our segmentation method and green bounding box according to validation data.

**Supplementary Table S1**. Overview of the number of image pairs and staining types used for validating histology registration. Serial sections were collected from 32 tissue samples. HE: hematoxylin and eosin; HES: hematoxylin, eosin, and saffron; IHC: immunohistochemistry; MTS: Masson’s trichrome stain.

**Supplementary Table S2**. Registration performance of HistoReg and GreedyFHist on directly neighboring serial sections. Registration accuracy is reported as the median of the median TRE (MM-TRE) and average of the median TRE (AM-TRE). AM-TRE metrics are reported with standard deviation (SD). TRE-based metrics are reported in µm and duration in seconds.

**Supplementary Table S3**. Overview of the number of image pairs and staining types used for validating group registration. Serial sections were taken from 32 tissue samples. HE: hematoxylin and eosin; HES: hematoxylin, eosin, and saffron; IHC: immunohistochemistry; MTS: Masson’s trichrome stain.

**Supplementary Table S4**. Comparison of different strategies for registration of distant serial sections. Registration accuracy is reported as the median of median TRE (MM-TRE) and average median TRE (AM-TRE). AM-TRE metrics are reported with standard deviation (SD). TRE-based metrics are reported in µm and duration in seconds.

**Supplementary Table S5**. Overview of integrated data used in proof-of-concept analysis. HE: hematoxylin and eosin; HES: hematoxylin, eosin, and saffron; MALDI MSI = matrix-assisted laser desorption ionization mass spectrometry imaging; ST: spatial transcriptomics.

**Supplementary Table S6**. Registration accuracy for spatial multi-omics integration. Spatial multi-omics integration was performed on 7 cancer-free prostate samples between stained images with spatial transcriptomics (ST) and stained images with MSI. Page 1 lists registration accuracy for the registration between ST section and MSI negative ion mode section (distance between sections: 50–100 µm), and page 2 lists the registration accuracy for the registration between ST section and MSI positive ion mode section (distance between sections: 40–80 µm).

**Supplementary Table S7**. Genes of citrate-spermine gene signature. Column 1 lists all genes defining the citrate-spermine gene signature. Column 2 lists all genes of the gene signature that were found in the spatial transcriptomics data.

**Supplementary Table S8**. Results of differential expression analysis between metabolites and CSGS separately for each sample. Significance of log2-fold change was evaluated using the Mann–Whitney *U* rank test on each metabolite and each sample separately.

**Supplementary Table S9**. Comparison of Spearman correlation between citrate-spermine gene signature (CSGS) and metabolites (citrate, zinc, spermine). Comparisons are performed with and without tissue matching for each metabolite and sample separately. ρ denotes the Spearman correlation coefficient, *n* the number of available data points, and *P* value to show the significance.

**Supplementary Table S10**. Comparison of correlations between CSGS and metabolites under the context of different integrated methods. The analyzed metabolites are citrate, spermine, and zinc. IntMatchHist: Spatial omics data were integrated, and only integrated spots from the same tissue type were considered. IntWithoutMatchHist: Spatial omics data were integrated, and all integrated spots were considered. ArtIntMatchHist: Spatial omics data were deliberately poorly integrated, and only spots with the same tissue type were considered. ArtIntWithoutMatchHist: Spatial omics data were deliberately poorly integrated, and all spots were considered. Correlations are reported as the Spearman coefficient ρ in addition to the *P* value and the distance between sections.

**Supplementary Table S11**. List of fixed parameters passed to Greedy during registration.

**Supplementary Table S12**. Averaged cross-validation results for YOLO8-based tissue segmentation and Otsu segmentation. Results are reported with mean and standard deviation.

**Supplementary Table S13**. Leave-one-stain-out cross-validation separated for each staining method in the test set. The staining methods are separated into hematoxylin and eosin (H&E); hematoxylin, erythrosine, and saffron (HES); Masson’s trichome staining (MTS); and immunohistochemistry (IHC).

**Supplementary Methods**. Description of manual landmark-based registration and description of immunohistochemistry staining of LPS and LTA.

**Supplementary Results**. Evaluation of YOLO8-based tissue segmentation.

## Abbreviations

AM TRE: average of the median target registration error; ArtIntMatchHist: integrated spots from the artificially integrated dataset with matching tissue type across all serial sections; ArtIntWithoutMatchHist: integrated spots from the artificially integrated dataset; CSGS: citrate-spermine gene signature; DHB: 2,5-dihydroxybenzoic acid; FFPE: formalin-fixed, paraffin-embedded; HE: hematoxylin and eosin; HES: hematoxylin, erythrosine, and saffron; IHC: immunohistochemistry; IntMatchHist: integrated spots from spatial transcriptomics and mass spectrometry data with matching tissue type across all serial sections; IntWithoutMatchHist: integrated spots from spatial transcriptomics and mass spectrometry data; LPS: lipopolysaccharides; LTA: lipoteichoic acid; MALDI-TOF MSI: matrix-assisted laser desorption/ionization time-of-flight mass spectrometry imaging; MIIT: Multi-omics Imaging Integration Toolset; MM TRE: median of the median target registration error; MRI: magnetic resonance imaging; MSI: mass spectrometry imaging; MTS: Masson’s trichrome staining; NCC: normalized cross-correlation; NEDC: N-(1-naphthyl) ethylenediamine dihydrochloride; ROI: region of interest; ssGSEA: single-sample gene set enrichment analysis; ST: Visium Spatial Gene Expression Array by 10x Genomics; TRE: target registration error.
