# Supplementary material for: Spatial integration of multi-omics data from serial sections using the novel Multi-Omics Imaging Integration Toolset
Source: Gigascience. 2025 May 14;14:giaf035. doi: 10.1093/gigascience/giaf035 (PMC12077394; doi:10.1093/gigascience/giaf035)
Supplement: giaf035_Supplemental_Files [file giaf035_supplemental_files.zip › Supplementary Material_Supplementary Figures.docx]

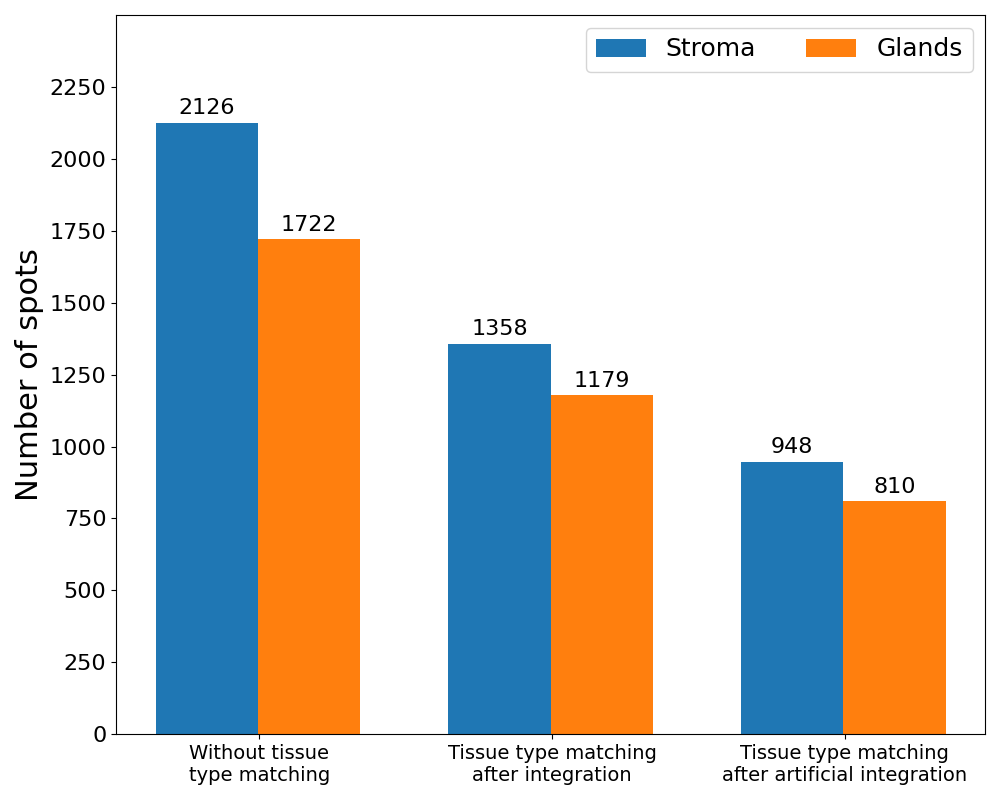


**Supplementary Figure 1.** Number of spots classified as glands or stroma with and without tissue type matching as well as after normal and artificial integration.


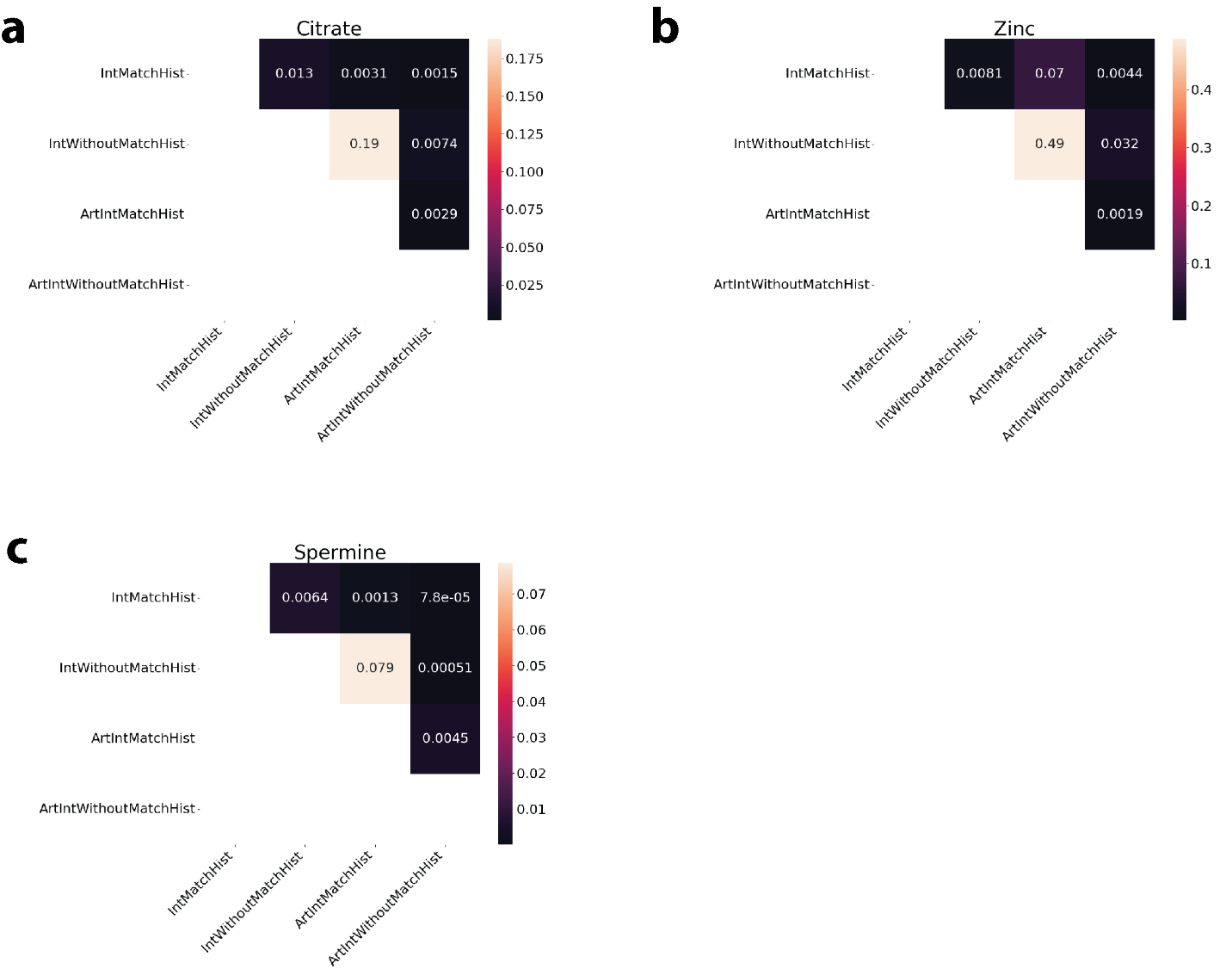
**Supplementary Figure 2.** a,b,c shows p-values for testing whether the difference of spearman correlation between two datasets is significant.


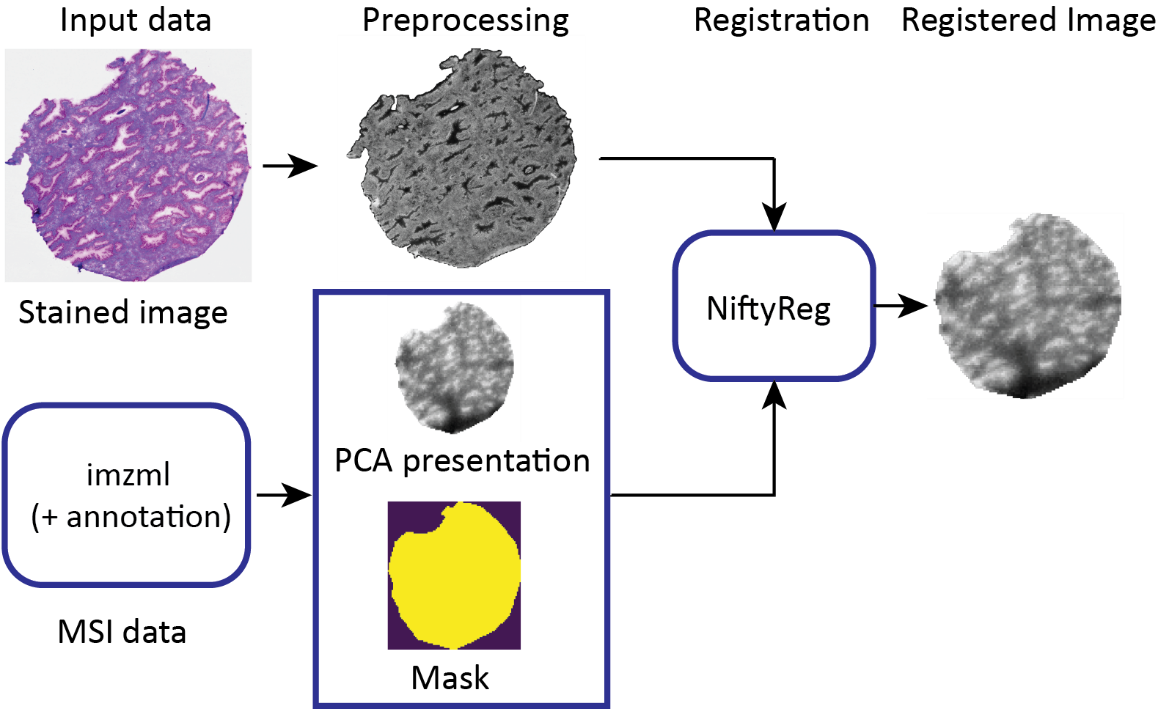


**Supplementary Figure 3.** Stained image is segmentation from background region. For the imzml file a feature image is derived based on the PCA decomposition. Then both images are padded symmetrically to a uniform shape and registered using NiftyReg. The resulting transformation is applied on the imzml data.


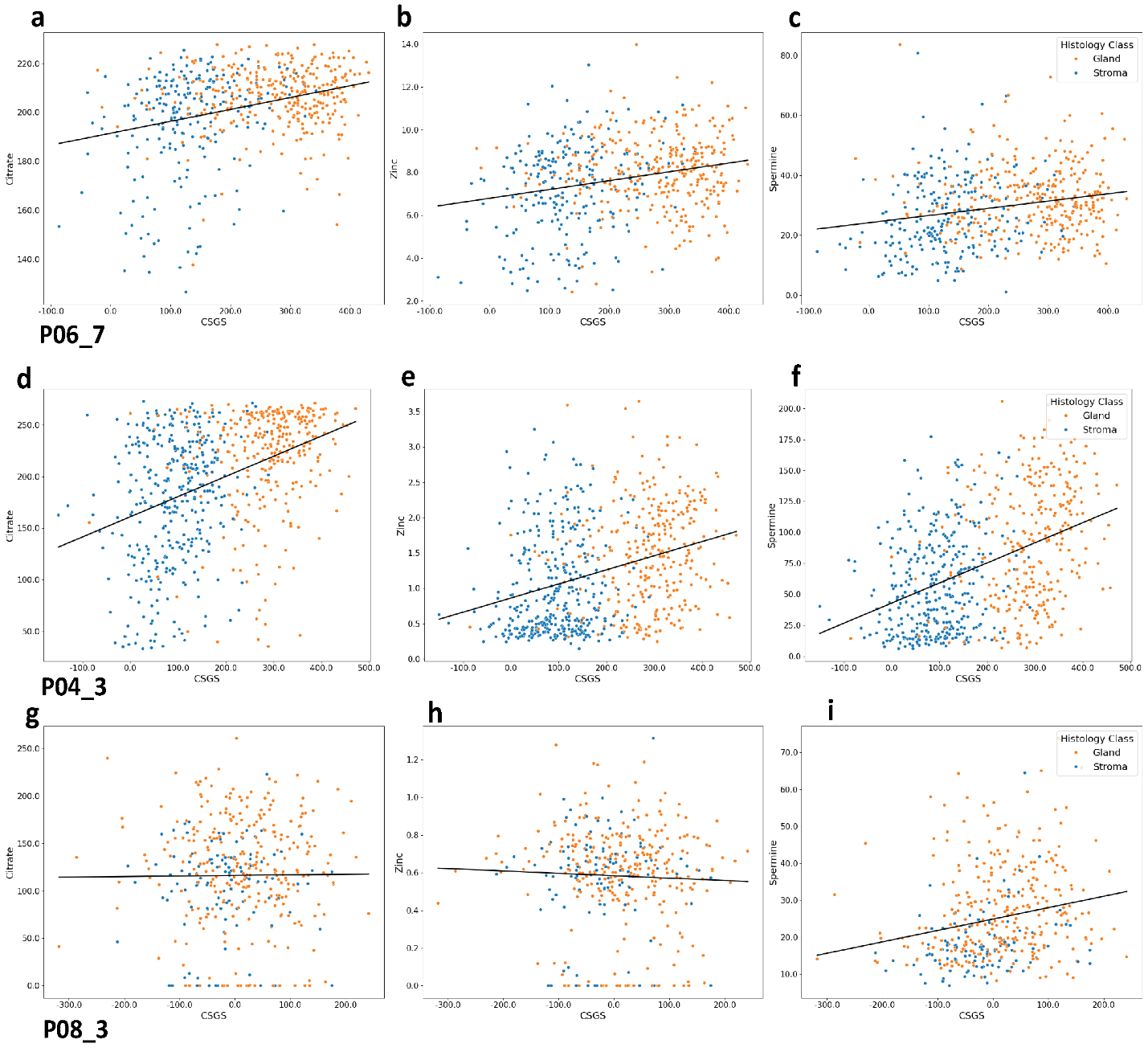


**Supplementary Figure 4.** Citrate (a, f, g), zinc (b, e, h), and spermine (c, f, i) levels plotted against CSGS score for samples P06_07 (a, b, c), P04_3 (d, e, f) and P08_3 (g, h, i) for integrated spots colored according to tissue type. Linear regression lines are shown.


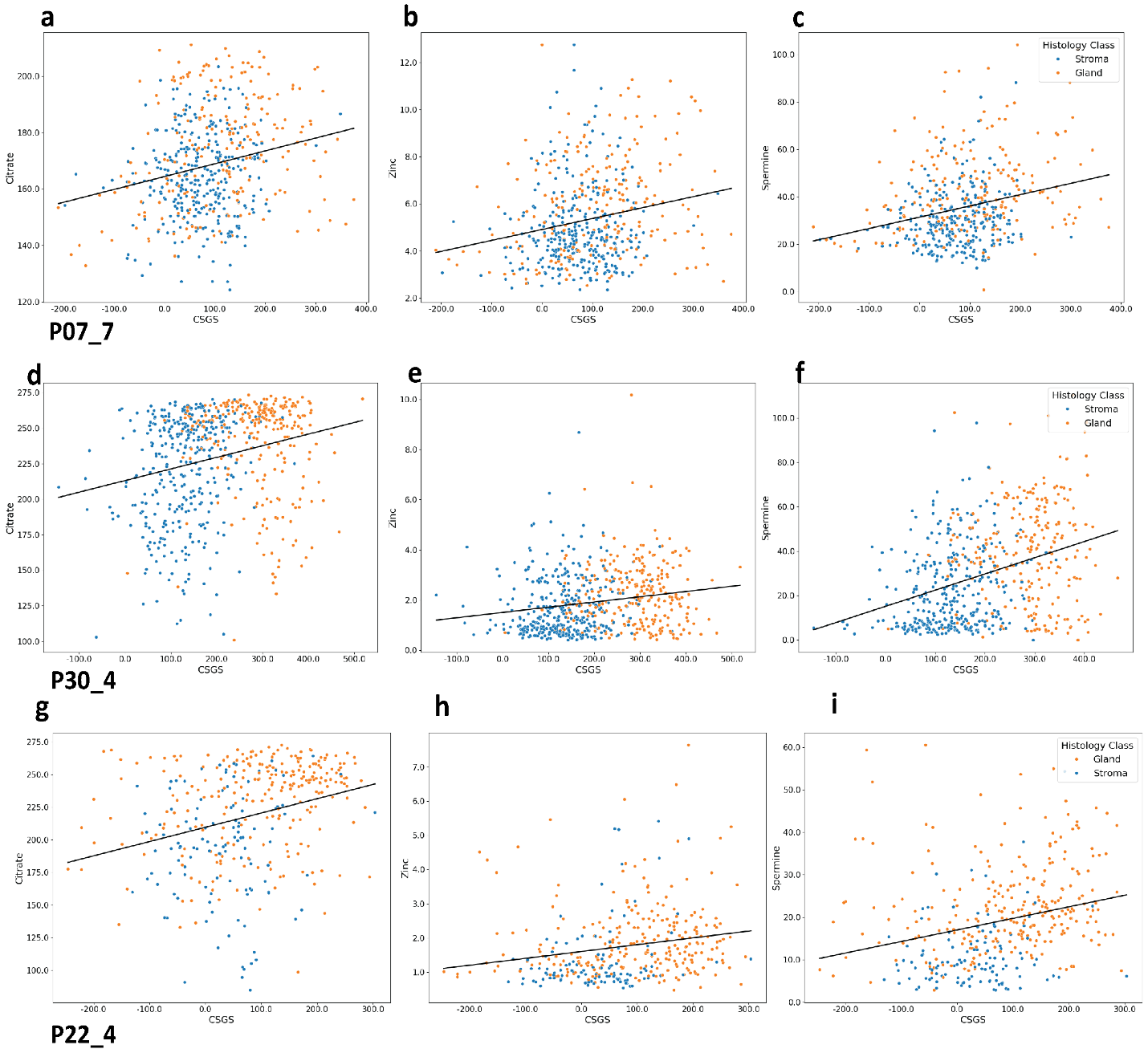


**Supplementary Figure 5.** Citrate (a, f, g), zinc (b, e, h), and spermine (c, f, i) levels plotted against CSGS score for samples P07_07 (a, b, c), P30_4 (d, e, f) and P22_4 (g, h, i) for integrated spots colored according to tissue type. Linear regression lines are shown.


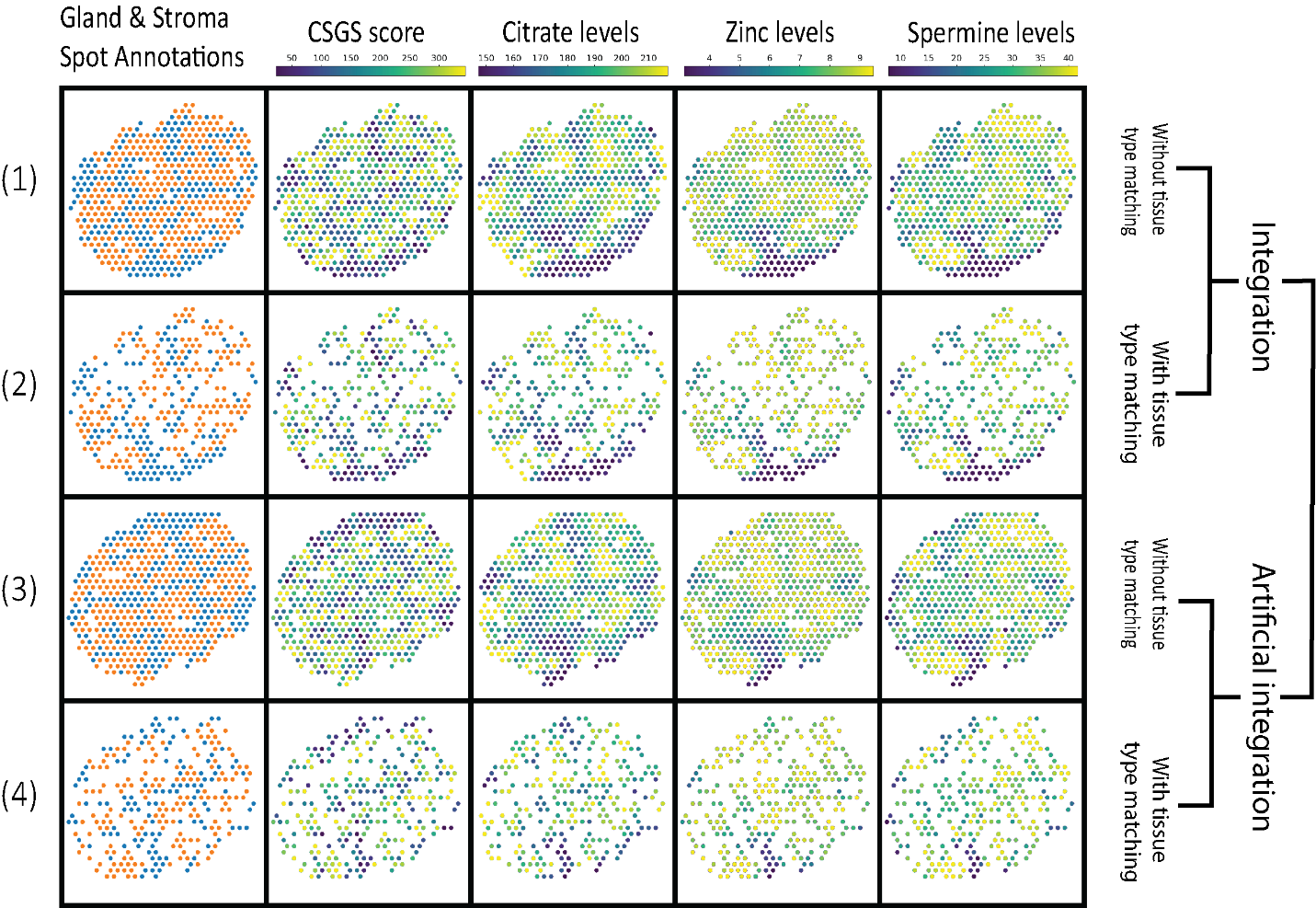


**Supplementary Figure 6.** Spot-wise distribution of gland and stroma, CSGS, citrate, zinc, and spermine for four different datasets (sample P06_7).


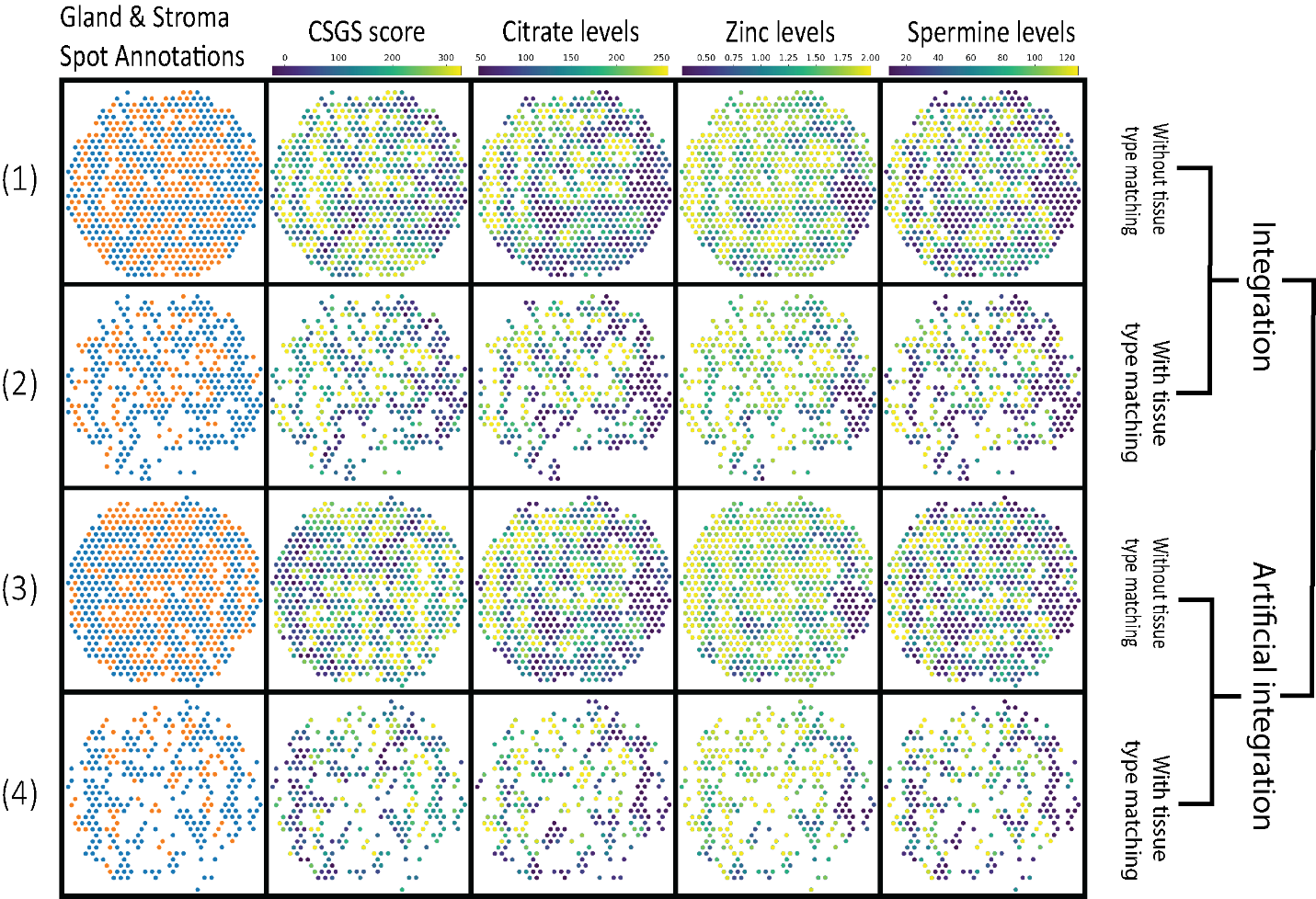
**Supplementary Figure 7.** Spot-wise distribution of gland and stroma, CSGS, citrate, zinc, and spermine for four different datasets (sample P04_3).


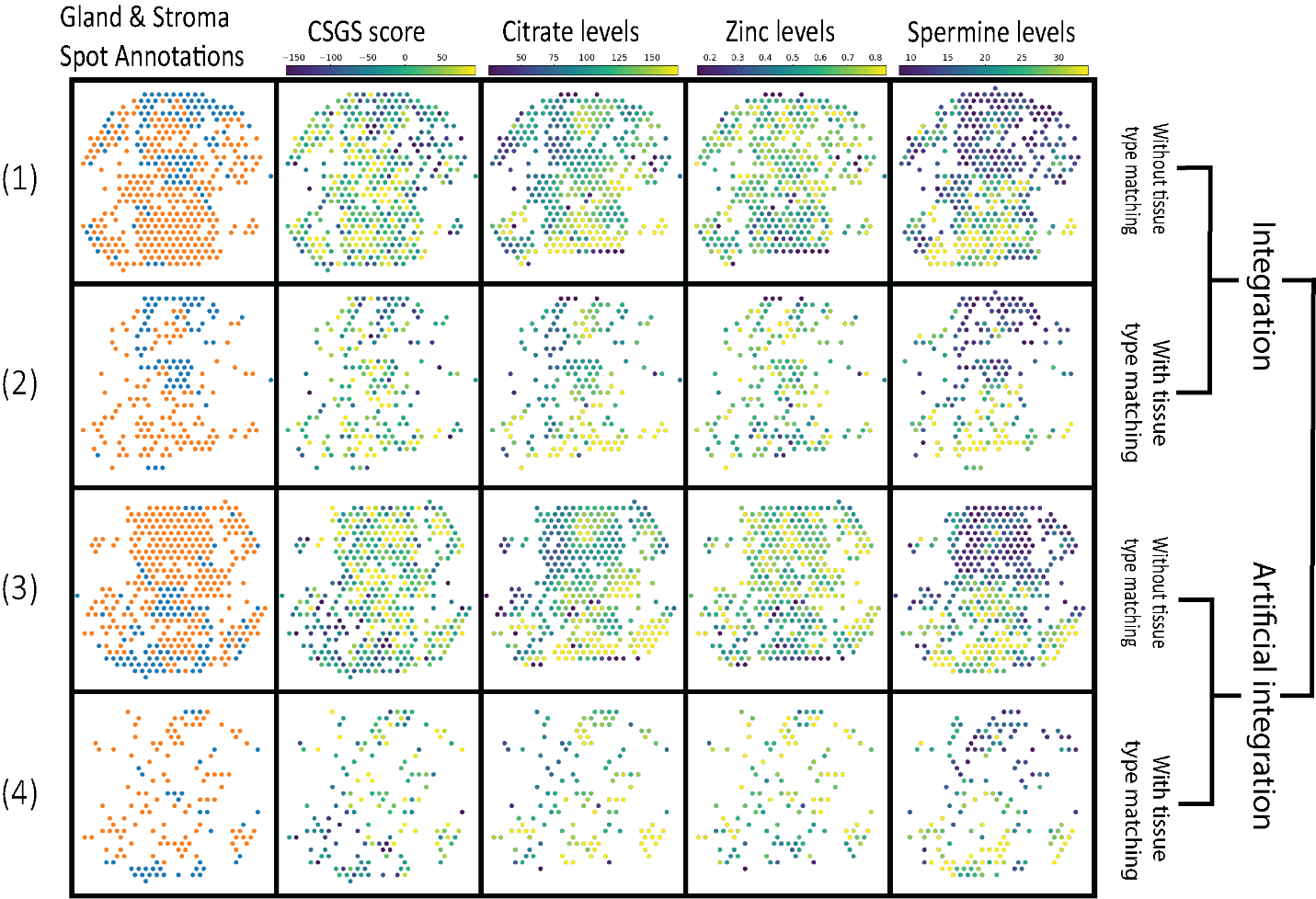
**Supplementary Figure 8.** Spot-wise distribution of gland and stroma, CSGS, citrate, zinc, and spermine for four different datasets (sample P08_3).


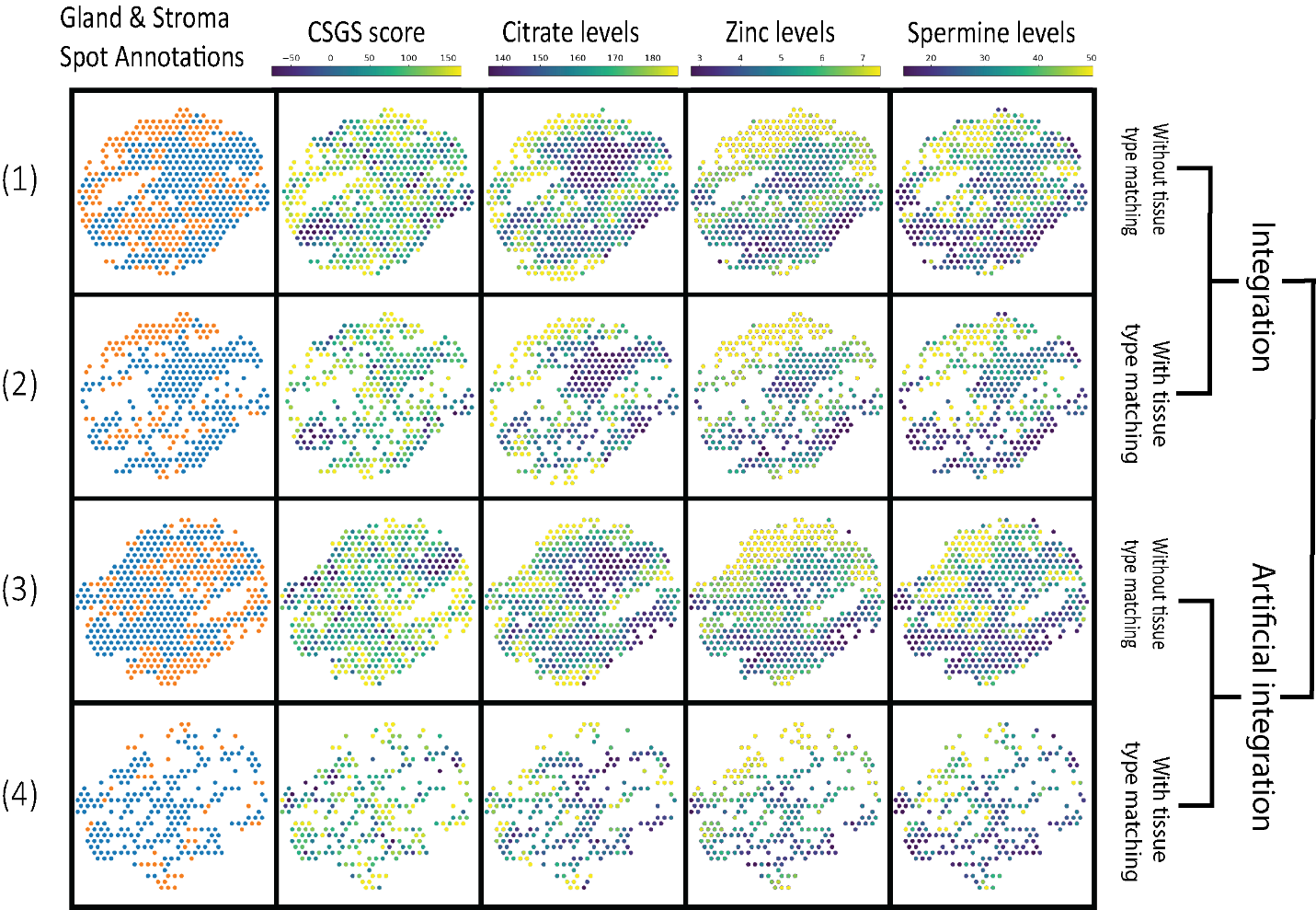
**Supplementary Figure 9.** Spot-wise distribution of gland and stroma, CSGS, citrate, zinc, and spermine for four different datasets (sample P07_7).


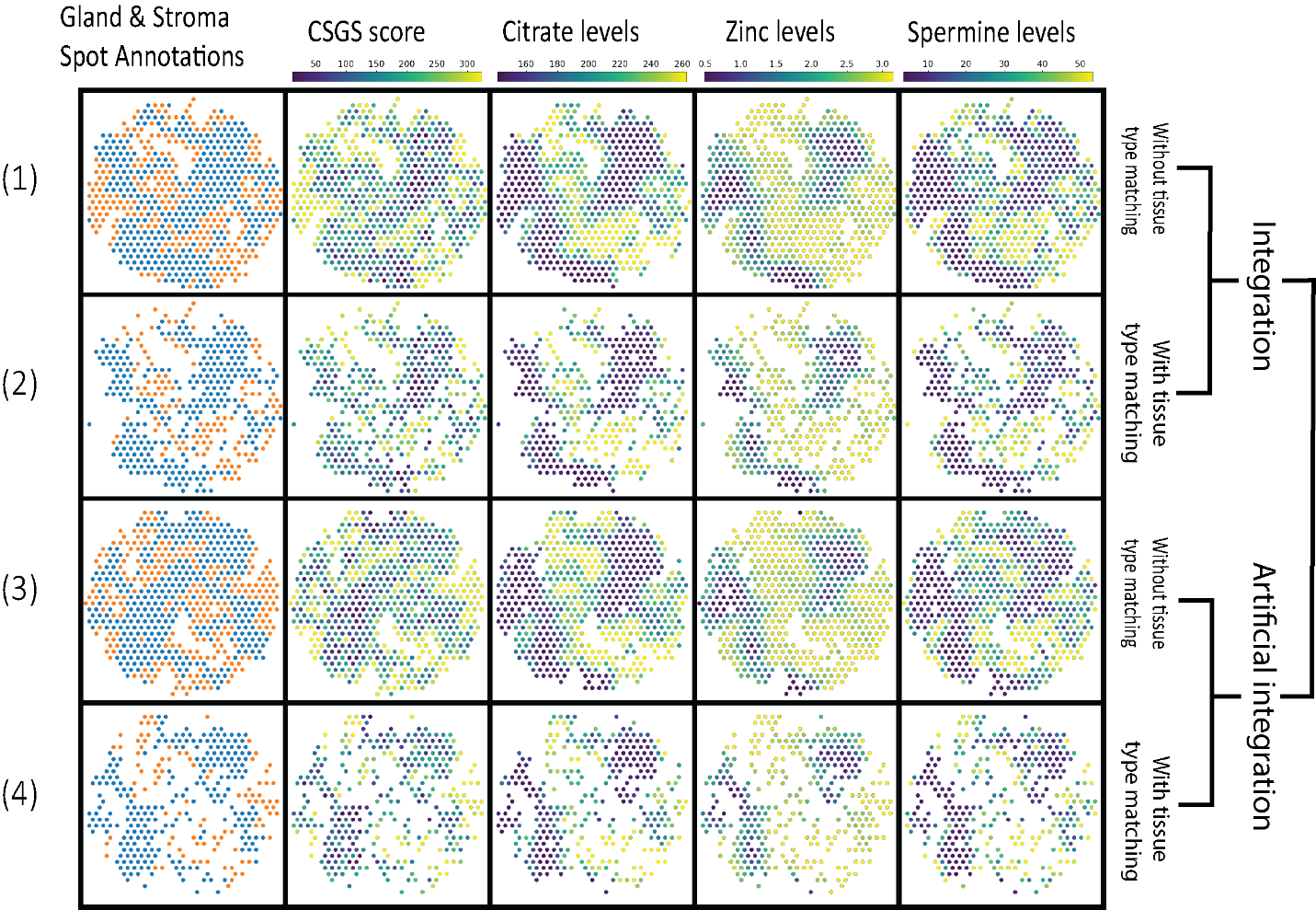


**Supplementary Figure 10.** Spot-wise distribution of gland and stroma, CSGS, citrate, zinc, and spermine for four different datasets (sample P30_4).


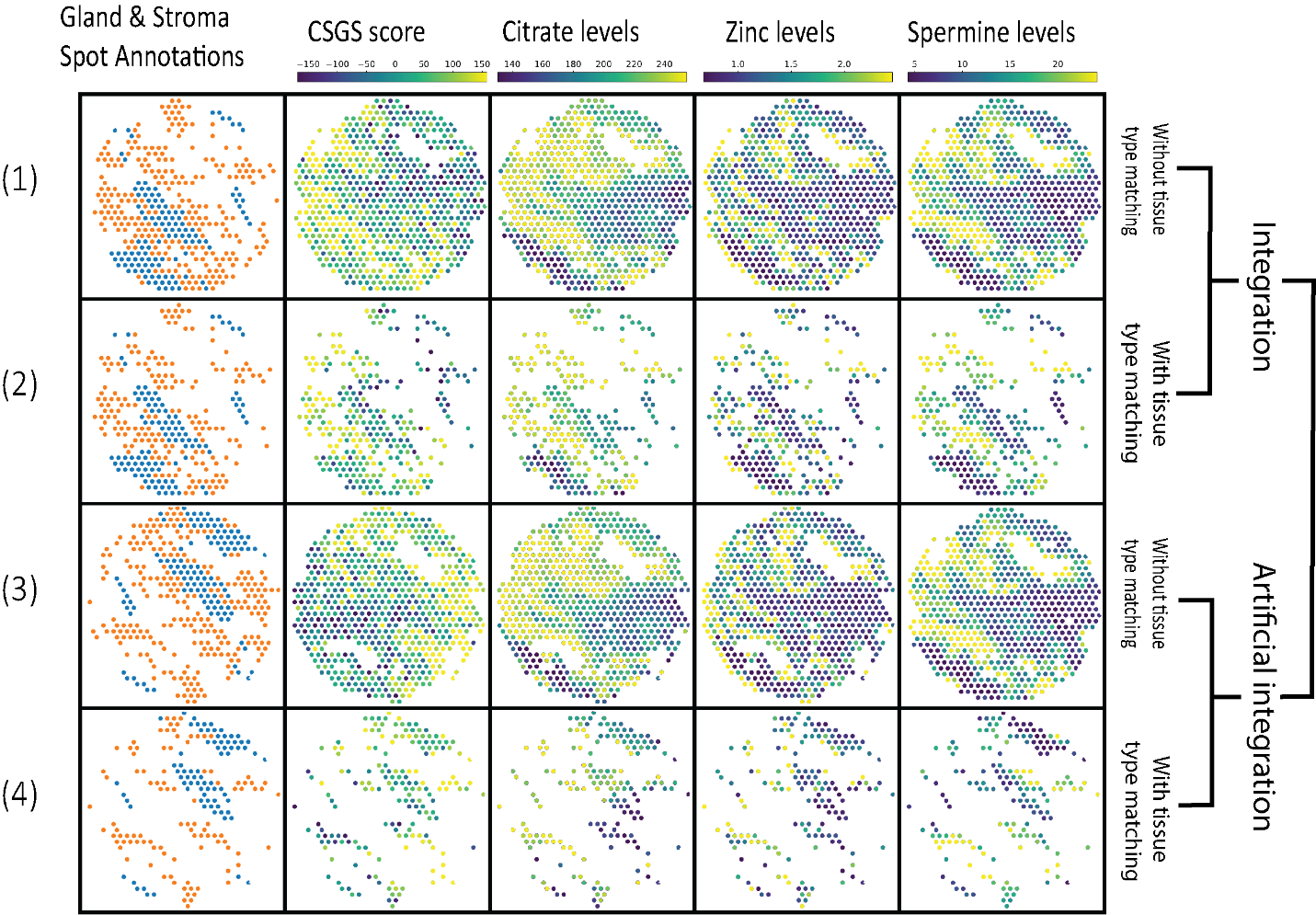
 **Supplementary Figure 11.** Spot-wise distribution of gland and stroma, CSGS, citrate, zinc, and spermine for four different datasets (sample P22_4).


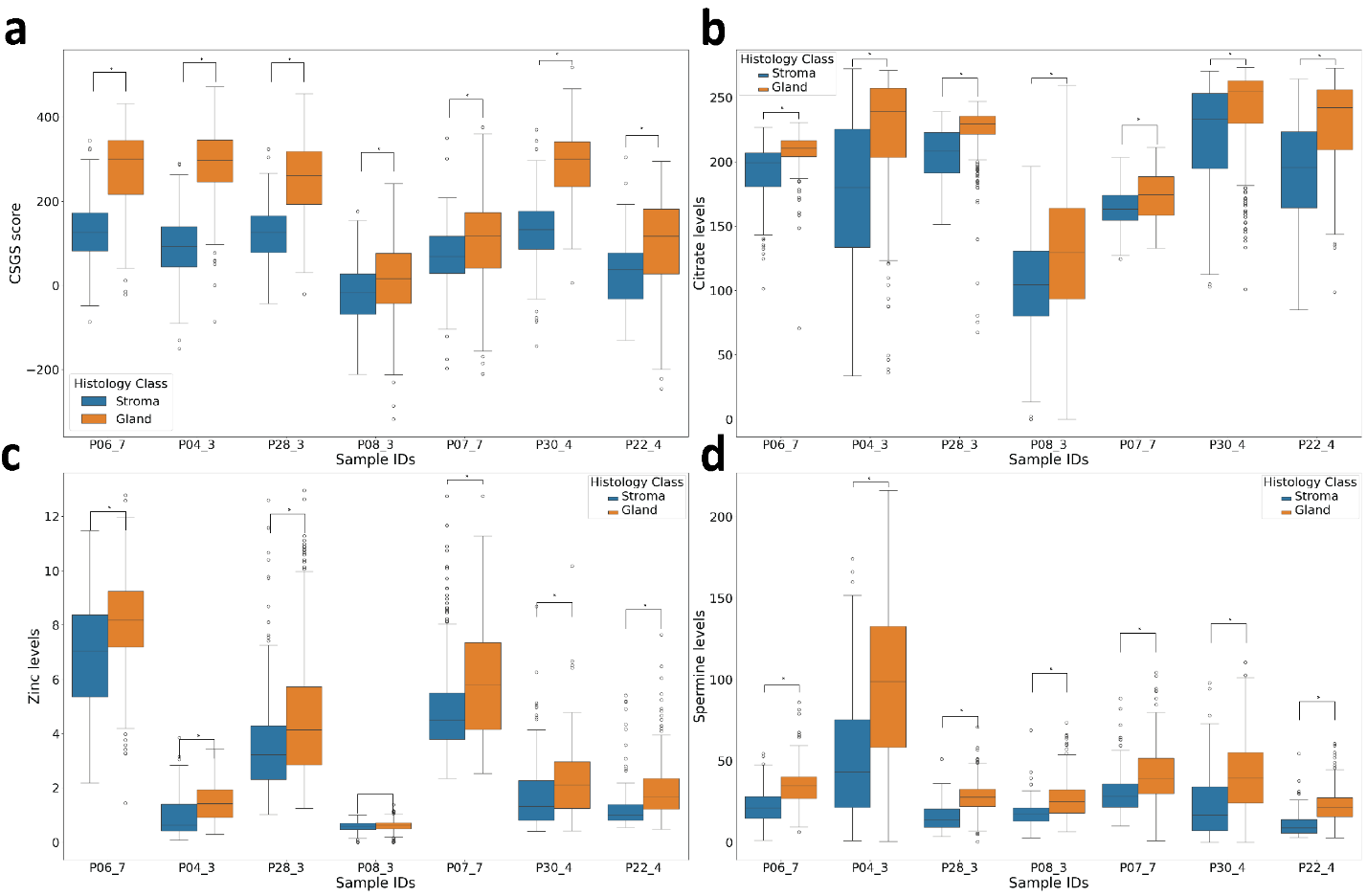


**Supplementary Figure 12.** Sample-wise gene scores and metabolite levels in stroma and gland spots for a) GSCS, b) citrate, c) zinc, and d) spermine.


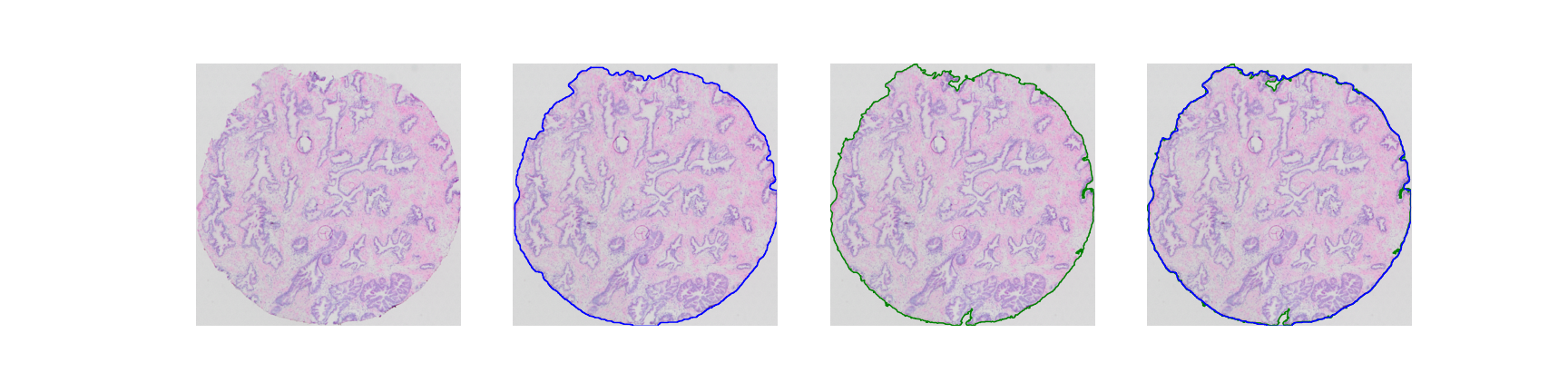


**Supplementary Figure 13. Validation of bounding box segmentation of stained image.** Blue highlights our segmentation method, green bounding box according to validation data.


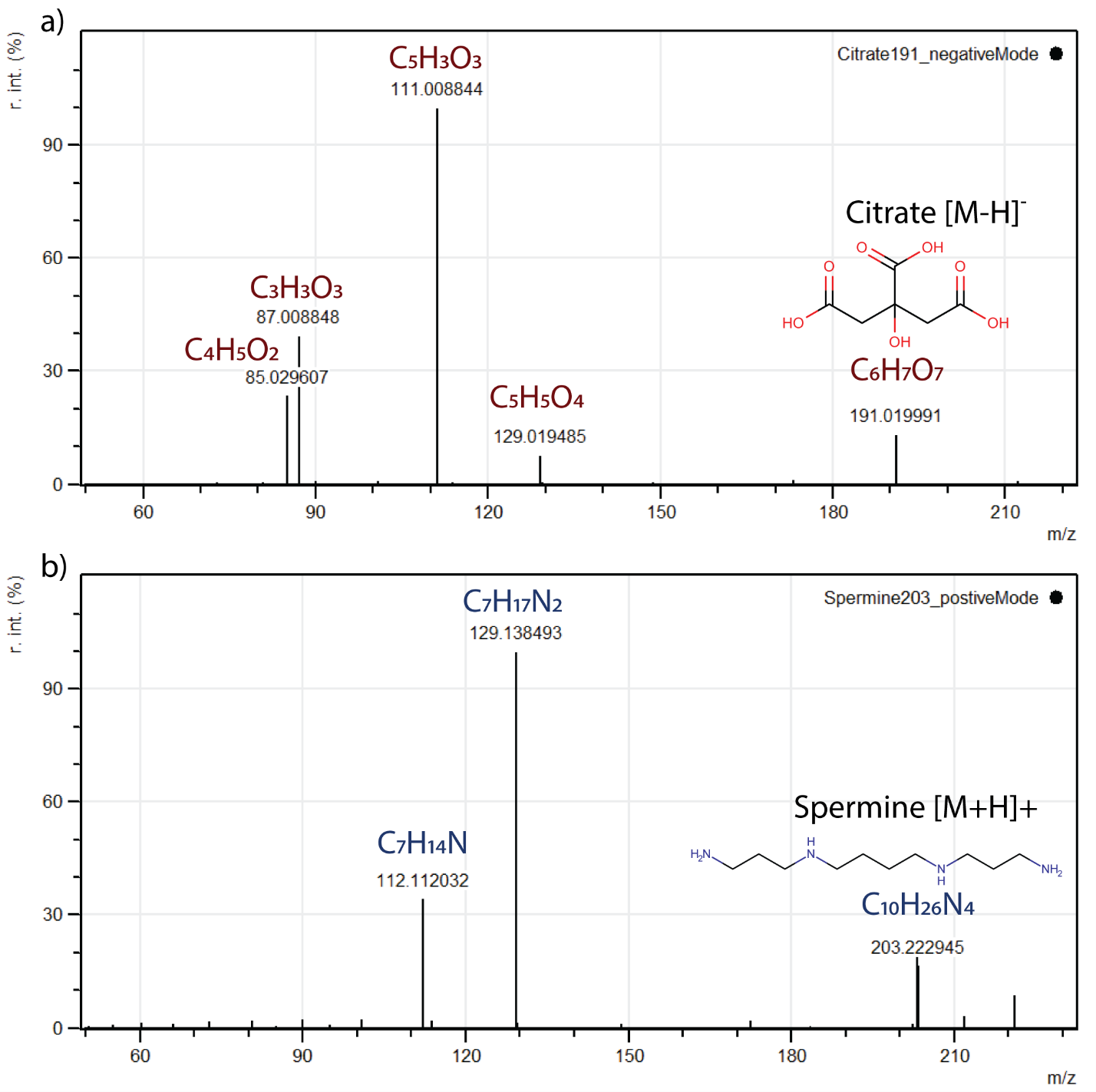


**Supplementary Figure 14. Tandem mass spectrometry for metabolite identification.** A) Mean on-tissue MALDI MS/MS spectrum of m/z 191.02 in negative ion mode identified as citrate [M-H]^-^ and of b) *m/z* 203.2 in positive ion mode identified as spermine [M+H]^+^.
