# Supplementary material for: Spatial integration of multi-omics data from serial sections using the novel Multi-Omics Imaging Integration Toolset
Source: Gigascience. 2025 May 14;14:giaf035. doi: 10.1093/gigascience/giaf035 (PMC12077394; doi:10.1093/gigascience/giaf035)
Supplement: giaf035_Authors_Response_To_Reviewer_Comments_Original_Submission [file giaf035_authors_response_to_reviewer_comments_original_submission.pdf]

Author's Response To Reviewer Comments

Dear reviewers and editor,
We appreciate the chance to send in an improved and revised version of our manuscript to GigaScience. We thank the reviewers for taking their time to read and provide both positive and constructive feedback on our work.

Reviewer #1: Wess et al reports a Python framework, MIIT (Multi-Omics Imaging Integration Toolset), for integrating spatially resolved multi-omics data. Multi-omics imaging represents a pivotal approach for systems molecular biology and biomarker discovery. This method introduces a timely and valuable tool to advance the field. However, in my opinion, this paper still has some issues that need to be addressed before consideration for publication.

Revision 1:

Cancer tissue exhibits significant heterogeneity effects, in this study, different molecular information stems from different tissue sections, this means from different cells as the tissue section is at 10 um thickness, almost the diameter of the cells. Please highlight the meaningful of co-registration information if they are obtained from different cell layers. In particular, for the datasets of spatial transcriptomics and MSI, the experiments were conducted on serial sections with an axial sectioning distance of 40 to 100 µm. This means that the mRNA and metabolites originate from different cells, raising questions about how integrating these two datasets can provide meaningful insights.

Answer:

With a distance of 80-100 µm between the tissue sections, RNA and metabolite measurements will stem from different cells. However, we focus on analyzing prostate cancer on a tissue compartment level and not on a single-cell level. The resolution of both data types used in our study (ST: 55 um radius and MSI: 30 um pixel size) typically encompass multiple cells (with potentially different cell types), thereby mixing signals. However, in prostate cancer we observe that cells that are close to each other within the same tissue type, exhibit similar patterns. We can see this in the proof of concept analysis of the citrate spermine gene signature (CSGC) and the metabolites (Citrate, spermine, zinc) (Figure 6b-d), where no significant difference was found between close and distant sections (p values: citrate -CSGS: 0.443; zinc – CSGS: 0.412; spermine – CSGS: 0.275, Welsh test) and we observe correlation between CSGS and the metabolites. Four samples (P06_7, P04_3, P08_3, P07_7) were extracted at a distance of 80 – 100 µm and 3 samples (P28_3, P30_4, P22_4) were taken at a distance of 40-50 µm. We tested whether the spearman correlations for the 2 groups for all 3 metabolites (i.e. citrate – CSGS, zinc – CSGS, spermine -CSGS) were significantly different. Additionally, we highlight that correlations are reduced between ST and MSI section if cells are too far apart by analyzing integrated data with a deliberately wrong registration. In the "artificial" integration we deliberately rotated the registered image by 180 degrees and proceeded with the integration as described (light orange) against correctly integrated data (darker orange) (Figure 6b-d). Correlations were lower in almost every case when comparing correlations and the number of significant correlations. This shows that cells in the same neighborhood do produce similar biological signals. Therefore, studying tissue types within their microenvironment will show valuable biological insight.
We have added the following paragraph on page 16 (bottom of page) – 17 (top of page):
"At a certain distance in the z-plane, the biological differences between two sections become significant, making it more practical to analyze each section separately rather than integrating the data. The maximum distance at which two tissue sections can still provide valuable data integration with MIIT should be determined by the user, based on the specific tissue being analyzed and the research question at hand. "

Revision 2:

The multi-omics imaging integration toolset is based on the GreedyFHist, a non-rigid registration algorithm. I suggest including more details about this algorithm and highlight the difference compared to previously reported non-rigid image co-registration algorithm.

Answer:
For more details on the GreedyFHist registration algorithm, we refer to the method section on page 33 ("Pairwise Tissue Registration – GreedyFHist"). We have included more details on how our registration method differs from others in the discussion on page 13 and 14:

Page 13 (last paragraph):
"Current algorithms tend to utilize computationally intensive deep learning methods for computing the registration, whereas we have opted for a lightweight algorithm that can run efficiently on a CPU architecture. GreedyFHist shares similarities with HistoReg, another state-of-the-art registration algorithm"
Page 14 (upper half of page):
GreedyFHist can process common formats in bioimaging analysis such as ome.tiff and geojson and apply transformations to various image and pointset data (e.g. simple coordinates and geojson). While several registration algorithms for histologically stained images have been proposed in recent years, only few have made their code available7,9, offer built-in support for processing of bioimaging files types, and have the functionality to apply the computed transformation to various image (e.g. single- and multichannel images and annotations) and pointset data (e.g. coordinate data, geojson) which are necessary for spatial multi-omics integration. Through these features, GreedyFHist interfaces with the well-known bioimaging software such as QuPath and ImageJ and is available as a separate software package where it can be used via command-line and interactively via API.

Revision 3:
The author should demonstrate the accuracy of background segmentation, it concerns certain low signal sample area would be removed in the denoising step.

Answer:
We definitely acknowledge the importance of evaluating the background segmentation, but due to space limit within the manuscript, we chose to focus more directly on the image registration and spatial multi-omics integration and described the accuracy in the supplementary (see document Supplementary Results, page 1, "Evaluation of tissue segmentation") with the following metrics (averaged with k-fold cross-validation with k =5): Hausdorff distance of 69.159 um (std: 54.434), DICE coefficient: 0.979, recall: 0.997; precision: 0.982). We further added a sentence in the manuscript on page 8 (second paragraph) that we hope will make the accuracy results more visible in the main text and where interested readers can be referred to the supplementary:
"The evaluation of our tissue segmentation algorithm demonstrated a higher segmentation accuracy than an alternative based on Otsu's thresholding (Hausdorff distance: 69.159 µm (our method) vs. 140.814 µm (Otsu); Supplementary Results). "

Revision 4:
What is the criterion to define the background region and sample region in the background segmentation.

Answer:
The criterion for defining the background region is based on a lightweight deep learning trained model based on the YOLO8 architecture for performing segmentation during registration. We used background segmentation to separate tissue area from non-tissue area and referred to non-tissue area as background. The model was trained on stained histology images containing four types of stains: HE, HES, MTS, IHC. For annotating the training data, we first use a pixel thresholder to generate initial masks. The threshold was adjusted for each image such that the tissue mask was approximately correct. Then we manually adjusted each initial mask to correct areas where the pixel thresholder classified the tissue wrongly. We have changed the heading "Preprocessing of stained images" to "Preprocessing of stained histology images and generation of tissue masks for segmentation training" page 22 (second paragraph):

“Preprocessing of stained histology images and generation of tissue masks for segmentation training. Tissue masks that separate tissue and non-tissue area (i.e. background) were used as training data for training our YOLO8-based segmentation model. They were created in QuPath (v 0.4.4). To speed up the process of annotating tissue area in histology images, we used a pixel thresholder to generate initial masks which were then manually corrected for misclassified areas. The pixel thresholder “Create Objects” function (options: Minimum object size: 40000 µm2; Minimum whole size: 1000 µm2) was used on the average intensity value of each pixel after applying a Gaussian filter (sigma: 1). The thresholds for HE, HES, and MTS were set between 208 and 215, for IHC the threshold was set between 218 and 225 due to lower staining intensity. The threshold was manually adjusted on each tissue image if necessary. Misclassified areas (e.g. due to noise in image or low staining intensity) were manually corrected using QuPath’s annotation tools. Stained images, tissue masks, and histopathology were exported using a groovy scrip to tiff images. The resolution for each image was set to 1 pixel per µm.”

Revision 5:
In the Method section, more details need to be included in the spatial transcriptomics part, what spatial resolution of the 10x Genomics was used.

Answer:
We have added information about the product codes for each tool and kit used in generating spatial transcriptomics data on page 20 - 21:

Visium Spatial Gene Expression Slide & Reagent kit (10X Genomics, product code: 1000184), Visium Spatial Tissue Optimization Slide & Reagent kit (10X Genomics, product code 1000193), QuantStudio™ 5 Real-Time PCR System (Thermo Fisher, product code A34322), Illumina NextSeq 500 instrument (SY-415-1001, Illumina®, San Diego, USA) using the NextSeq 500/550 High Output kit v2.5 (150 cycles; product code 20024907)

We have also added information about the resolution of the spatial transcriptomics data at the end of the paragraph on page 21 (first paragraph):

“Each distinct coverage area (ST-spot) has a radius of 55 µm and the distance between the center of each ST-spot is 100 µm.”

Revision 6:
As the MALDI resolution is 30 um, how the pixel alignment of the ST and MSI data if their spatial resolution is different.

Answer:
We address this issue in the description of MIIT in the results and method section, namely in the preprocessing and fusion steps: In preprocessing we perform a grid projection in which we map each spatial data point (i.e. each ST-spot and msi-pixel) onto the image space of the stained histology image (which we refer to as the reference matrix). For instance, that means that for a stained histology image of resolution 1 um per pixel, a msi-pixel of size 30 um2 is referenced on 900 pixels in the reference matrix. This allows for applying nonrigid registration and for accurate transformations from the moving image space to the fixed image space. During the fusion step, we can use the reference matrix to compute the overlap between the reference matrices for ST and MSI. Because each msi-pixel is upscaled in the reference matrix to a resolution of 1 um per pixel, we can compute how much of each MSI-pixel overlaps with each ST-spot (see also: Figure 3c “Spatial mapping of ST references to MSI references"). In that way we can fuse together MSI and ST data with different spatial resolution. We made some changes to the description of the preprocessing and transformation of the spatial integration on pages 27 - 29:

Preprocessing (page 27 - 28):
“In this format, the coverage of each data point in the image space of the stained histology is computed. We initialize an empty matrix (reference matrix) with the same resolution as the stained histology. Then, for each pixel that matches the coverage of a spatial data point, we place a reference to the matching

spatial data point. This ensures that spatial omics data can be transformed accurately during the non-rigid registration and allows fine-grained fusion between spatial omics datapoints. In this format, each spatial omics datapoint is represented by a reference and projected onto a matrix with the same resolution as the stained image."

Fusion (page 29; lower half of paragraph):

"Due to performing the grid projection in step 1, we can use the overlapping area between the reference matrices to calculate the fraction that each MSI-pixel contributes to the shared area with each ST-spot."

Revision 7:
In the MALDI-MSI of prostate tissue, on tissue MS/MS data it is missing to confirm the identification of target analytes of citrate, ZnCl3-, and spermine.

Answer:
We acknowledge that the background for mass identification was not sufficiently described, and we thank the reviewer for noticing. To verify analyte identities on citrate, ZnCl3- and spermine, we measured on-tissue MS/MS and accurate mass by a high-mass resolution MALDI-Orbitrap instrument as previously described in a former publication (see references below). We have provided MS/MS spectra of citrate and spermine in Supplementary Figure 14, while data for identifying zinc is accessible in Andersen et al. 2020. Since these previous experiments were performed on tissue sections with identical characteristics and with using the same matrices (NEDC and DHB) as in this current study, the analyte identifications were considered valid. We have added the following text in the method section describing the MALDI-MSI experiments on page 21 (bottom of page) – 22 (top of page):

"We selected masses that we previously identified on a similar fresh frozen prostate tissue dataset where on-tissue tandem MS and accurate mass acquisition with a high mass resolution instrument (MALDI-Orbitrap) were used to confirm analyte identity (Supplementary Figure 14). Zinc was detected in negative ion mode in the form of ZnCl3- and was identified through accurate mass and the isotopic pattern as described by Andersen et al. The mass identification in our previous studies was performed on prostate tissue sections retrieved and stored the same way, and covered with the same matrices, NEDC and DHB for negative and positive mode, respectively.

References:
Andersen, M. K. et al. Spatial differentiation of metabolism in prostate cancer tissue by MALDI-TOF MSI. Cancer Metab. 9, 1–13 (2021).
Andersen, M. K. et al. Simultaneous detection of zinc and its pathway metabolites using MALDI MS imaging of prostate tissue. Anal. Chem. 92, 3171–3179 (2020)."

Reviewer #2: Overview:
In this paper, the authors present the Multi-Omics Imaging Integration Toolset, which is a python framework for integration multiple spatial omics datatypes. To facilitate this, they also development a registration method (GreedyFHist) for jointly analyzing sequential tissue layers that have undergone different types of staining/phenotyping regimens. The method validation was done on a 244 fresh-frozen prostrate tissue sections. The highly detailed methods and results section is well appreciated and helps fully contextualize the significance of the study. The definitions of study-specific terms mentioned throughout the paper at the beginning are also appreciated.

Data and Code Availability:
Detailed code, tutorials and associated instructions have been made available for use by the public, which is appreciated. All systems requirements have also been explicitly laid out for ease of installation and use. The workflow examples provided are quite detailed; however, a more extensive codebase with stepwise explanations within the code will be appreciated.

Answer:
We have now released documentation for both tools and added missing documentation to all functions: https://greedyfhist.readthedocs.io/en/latest/ and https://miit.readthedocs.io/en/latest/.
Data has not been made available publicly, except for the raw and processed spatial transcriptomics data; however, detailed and explicit instructions have been provided on data access, keeping in mind local regulations.

Revisions:
Major Revisions:

Revision: 1. In recent years, a lot of other platforms, both free and paid, tend to support registration across multiple slides. For example, HALO has a registration feature available as well, along with a host of other open-source datatypes. In that regard, how is your platform different?

Answer:
See question from reviewer 1. Please note that we did not include HALO as we focus on using open-source solutions. Technically it might be possible to add HALO to our integration platform, but we could not validate it as we do not have access to HALO.

Revision: 2. It is mentioned that downscaling occurs during the registration process in order to reduce runtime - how are nuances in features selected as registration landmarks preserved in such a case?

Answer:
Downscaling is common technique in image registration. Although it is possible that some nuanced landmarks are removed during the nonrigid registration due to downscaling, we noticed only a limited effect on the registration accuracy. Downsizing also removes subtle non-matching features between moving and fixed images (e.g. isolated cells, small color variances due to different staining).
We performed registration with three different resolutions and all 3 resolutions performed similarly as presented in the following table:

Registration mode / resolution	AM-TRE	MM-TRE
Nonrigid (1024)	37.463 (best)	21.025
Nonrigid (2048)	39.312	19.538 (best)
Tiling (tile size (2048; see explanation below)	38.165	20.257

We also added a tiling option for the nonrigid registration:
- Tiling allows to register images without downsizing at the cost of requiring more computation.
- In this mode, moving and fixed image are separated into smaller tiles (e.g. of size 2048x2048 each) which are then registered. After registration, resulting transformations are stitched together to one transformation of the size of the moving and fixed images.

We can see that the registration accuracy does not change considerably when using different resolutions. Please note, that we decided to use a resolution of 1024 as it achieved the best average median – target registration error.

Please note, that since the original submission we have done some minor bug fixes and some minor parameter optimization on the MIIT framework and GreedyFHist. After that, we noticed slight improvements in the registration accuracy which is why we have updated the evaluation of the registration according to the table above and page 8-9 in the manuscript.

Nonetheless, in some instances a more nuanced registration of ROIs can be required. In that case, GreedyFHist has the option to run an additional registration using custom segmentation masks on ROIs

between moving and fixed image. These ROIs are typically smaller and allow for more image details to be captured. We have changed the text on page 6 (bottom of page) to highlight this option better:

“Furthermore, GreedyFHist has the option to fine-tune regions of interest in the histology images by supplying custom segmentation masks.”

Revision: 3. How is the fixed image determined in this case? The assumption would be that a standard H&E image is selected for this purpose- is that assumption, correct?

Answer:
For the registration, the fixed image can be chosen arbitrarily, because after the registration both moving and fixed image (and any additional spatial omics data and tissue annotations that is associated with either image) are spatially aligned.
Indeed, the assumption is correct that we chose the H&E stained image with ST data as the fixed image. We did this because more work on this section (e.g. analysis, annotations) had been performed prior to this study. However, in theory we could have also chosen the HES image (with the MSI data) as well.

Revision: 4. The authors have stated and justified their rationale for using the mentioned evaluation metrics in the paper. However, in the general image registration space, metrics such as the dice coefficient and jaccard index are commonly used and accepted. Is there a particular reason why these were not used as well? It would offer a more complete picture for the general user if these metrics were provided as well.

Answer:
Using DICE coefficient or Jaccard index as a metric would require annotating tissue regions of interest (such as cancerous area and glands) in addition to landmark annotations. We have annotated tissue areas that could be used to measure dice coefficient or jaccard index, but found it not feasible in our data due to several reasons:
- To set matching landmarks between serial images is less time-consuming than annotating areas, since that would require drawing accurate polygons along the tissue features (e.g. glands, stroma etc).
- Some of our tissue samples exhibit at least mild tissue damage. This is challenging because such missing tissue would result in a lower DICE coefficient. This is not something the algorithm can account for, but for landmarks not present, they can simply be removed.
- Changing tissue morphology in serial sections is an additional challenging factor for using metrics such as dice and the jaccard index. Tissue compartments will spatially alter their shape, including split into multiple compartments or merge with neighboring compartments across serial sections, and it is therefore difficult to track compartments across slides.

Revision: 5. The validation of registering distance neighboring sections is quite a valuable contribution, as the authors rightly stated that in many multi-omics experiments, this might be a necessity. However, when looking at tissue sections that are 80-100 microns apart, it is quite likely that the set of cells that one may be looking at on the x-y coordinate system may not be the same at all; in fact, for a highly heterogeneous/flexible piece of tissue, they might be completely different. In such a circumstance, how much value is there in registering these two sections together instead of, say, separately analyzing them and using alternative methods to combine the results downstream?

Answer:
See answer to question raised from reviewer 1, page 1-2.

Revision: 6. In the proof of concept presented in the paper, the authors mention using ST and MSI data for validating their framework. Have they also investigated ST integration with more commonly available datatypes such as IHC/mIF?

Answer:

In this paper, our main focus is the integration of ST and MSI (as well as the validation of our registration on differently stained tissue) and the release of the computational framework that we used to integrate data (MIIT). The addition of other imaging modalities such as the immunohistochemistry or multiplexed immunofluorescence are interesting, and we plan to add new imaging modalities in future projects in which utilize the MIIT framework. In the case of integrating mIF and ST we would follow the same procedure as described in the paper but replace MSI with mIF. In the transformation step we would then accumulate staining intensities over each ST spot.

Revision: 7. The work that the authors have put in to validate the registration and MIIT framework using different approaches (selecting spatially distant slides, integration using augmented/artificial data) is thorough. However, different tissue types bring in their own challenges, and thus validation of this framework on an external dataset would lend more credence to this much needed framework, especially in the era of increased multiomics analyses.

Answer:
We have attempted, but were unsuccessful in finding additional publicly available datasets that we could use for validation. While analysis of spatial transcriptomics and MSI from serial sections has been attempted before, the public data typically lacks the required stained histology images from serial sections.

Minor Revisions:
Revision: 1. Please ensure all typos/grammatical mistakes are corrected.

Answer:
Typos and grammatical errors are corrected by our best abilities.

Revision: 2. In the 'preprocessing of stained histology images', can more details be given on the thresholding process? It is also stated that the threshold is manually adjusted for each image if necessary - how is this determination done?

Answer:
Please see answers to the questions raised by reviewer 1, page 4-5.

Revision: 3. The headings/subheadings organizations within sections can be done in a more organized manner, in some parts it was challenging to determine the organization of sections/subsections.

Answer:
The sections, headings and subheadings are now organized highlighted as follows:

Section
Heading
Subheading

Furthermore, we have removed some redundant subheadings from the result section pages 6-8.

Revision: 4. Can some more details be given on the landmarks that were identified per image? Could some examples be provided on what these landmarks are, and how they remain consistence across tissue layers?

Answer:
We have made changes in the text to give more detail on how landmarks were chosen on page 26 (second paragraph):
"Histological landmarks were chosen based on visually distinct histological features that are present in adjacent tissue sections, such as distinct glandular shapes, gland and stroma formations, and high-density

clusters of cells, but also more nuanced image features, such as perforations in glandular structures. This ensured that both prominent and subtle image features were evaluated for registration performance. We aimed to annotate the same landmarks throughout serial sections and distribute them over the whole tissue area. If a landmark could not be located in an adjacent section (e.g. due to the disappearance of a gland, the tissue being too homogenous to identify the distinct feature, or disappearance due to tissue damage), a new landmark pair was set when possible to keep the total number of landmarks consistent."

Revision: 5. Currently, the way various samples are used for validating the GreedyFHist and MIIT frameworks are listed out in the paper is quite confusing. It would be appreciated if the authors can distinctly mention the number of samples out of the set of samples, and the associated stained slides are used for each.

Answer:
We have updated the description of the validation for the pairwise and groupwise registration validation of GreedyFHist, and the Proof of concept analysis of MIIT in the text and added new supplementary tables (Supplementary Tables 1,4,6):

Page 8 (third paragraph):
"First, we investigated the registration accuracy of GreedyFHist for the registration on adjacent serial sections. For our test data, we selected each pair of adjacent sections (n=7) from 32 samples (Figure 1). If at least one of the images in each pair was too damaged for accurate registration, we discarded the pair. This resulted in 212 pairs of adjacent serial sections (distance of 10 – 20 μm) consisting of 4 different types of staining (Supplementary Table 1)."

Page 9 (second paragraph):
"From 32 samples we collected each image pair with a distance of 5 sections (n=4) corresponding to 50 – 100 μm. Images that were too damaged for registration and images that GreedyFHist could only register poorly during the pairwise evaluation (median-TRE > 200 μm; Figure 4d) were not included resulting in 122 image pairs (Supplementary Table 3). "
Page 10 (last paragraph):
"For investigating the relation between CSGS and metabolite levels, we chose a subset of 7 cancer-free samples from 32 samples where each sample was taken from different prostate cancer patients."

Revision: 6. How were the annotations from the 3 annotators cross-validated?

Answer:
After the landmarks were placed initially, we handed annotators a set of images and landmarks that they had not worked on before. Then we validated the dataset by visual inspection.
We have added the text to make it clearer how annotation was performed on page 26 (bottom of page):
"After the initial placement of landmarks on serial sections, landmarks were cross-validated by visual inspection by a different annotator who had not worked on this set of serial sections. Though annotators mostly agreed on the placement of landmarks, a consensus was reached whenever annotators disagreed."

Further author notes:
Please note that in addition to the review we made the following changes:
- We have added a list of abbreviations to the end of the document as well as additional files.
- We have updated the submission to be in line with Gigascience submission guidelines.
- Figure legend are placed at the end of the manuscript and figures are provided separately.
- In the description of the MIIT algorithm we have changed the name of step 3 from "transformation" to "fusion", because it reflects better the merging of the ST spot and MSI pixels and the term "transformation" is also used when describing the image registration, which can lead to confusion.

We also have the possibility to provide a document with track changes.

Cookie Preference Center

We use cookies which are necessary to make our site work. We may also use additional cookies to analyze, improve and personalize our content and your digital experience. For more information, see our Cookie Policy.

You may choose not to allow some types of cookies. However, blocking some types may impact your experience of our site and the services we are able to offer. See the different category headings below to find out more or change your settings.

Allow all

Manage Consent Preferences

Strictly Necessary Cookies

Always active

These cookies are necessary for the website to function and cannot be switched off in our systems. They are usually only set in response to actions made by you which amount to a request for services, such as setting your privacy preferences, logging in or filling in forms. You can set your browser to block or alert you about these cookies, but some parts of the site will not then work. These cookies do not store any personally identifiable information.

Functional Cookies

These cookies enable the website to provide enhanced functionality and personalisation. They may be set by us or by third party providers whose services we have added to our pages. If you do not allow these cookies then some or all of these services may not function properly.

Performance Cookies

These cookies allow us to count visits and traffic sources so we can measure and improve the performance of our site. They help us to know which pages are the most and least popular and see how visitors move around the site.

Targeting Cookies

These cookies may be set through our site by our advertising partners. They may be used by those companies to build a profile of your interests and show you relevant adverts on other sites. If you do not allow these cookies, you will experience less targeted advertising.

Cookie List

Clear

Apply Cancel
Consent Leg.Interest

Confirm my choices

Powered by onetrust
