# Supplementary material for: Spatial integration of multi-omics data from serial sections using the novel Multi-Omics Imaging Integration Toolset
Source: Gigascience. 2025 May 14;14:giaf035. doi: 10.1093/gigascience/giaf035 (PMC12077394; doi:10.1093/gigascience/giaf035)
Supplement: giaf035_Authors_Response_To_Reviewer_Comments_Revision_1 [file giaf035_authors_response_to_reviewer_comments_revision_1.pdf]

Author's Response To Reviewer Comments

Dear Editor,

Thank you for the feedback. I have addressed all open points. Some minor changes and comments:

1) I added a section "ORCIDs" for all authors that have an ORCID.

2) I added the double dagger with the description "denotes equal contribution by senior author".

3) I decided to split the section "Availability of Source Code and Requirements" into two sub sections (one for MIIT, and one for GreedyFHist). Since RRIDs, bio.tools IDs, and reference to software heritage should be added for both tools it became a bit messy to have GreedyFHist only in sentence form.
Please note, that I added the reference to DOME_ML in the subsection for GreedyFHist, since this is where the ML model is primarily used and deposited via github.

4) I changed the format of the citations to the format requested in the citation guidelines.

I hope all of the minor revisions have been addressed.

Cookie Preference Center

We use cookies which are necessary to make our site work. We may also use additional cookies to analyze, improve and personalize our content and your digital experience. For more information, see our Cookie Policy.

You may choose not to allow some types of cookies. However, blocking some types may impact your experience of our site and the services we are able to offer. See the different category headings below to find out more or change your settings.

Allow all

Manage Consent Preferences

Strictly Necessary Cookies

Always active

These cookies are necessary for the website to function and cannot be switched off in our systems. They are usually only set in response to actions made by you which amount to a request for services, such as setting your privacy preferences, logging in or filling in forms. You can set your browser to block or alert you about these cookies, but some parts of the site will not then work. These cookies do not store any personally identifiable information.

Functional Cookies

These cookies enable the website to provide enhanced functionality and personalisation. They may be set by us or by third party providers whose services we have added to our pages. If you do not allow these cookies then some or all of these services may not function properly.

Performance Cookies

These cookies allow us to count visits and traffic sources so we can measure and improve the performance of our site. They help us to know which pages are the most and least popular and see how visitors move around the site.

Targeting Cookies

These cookies may be set through our site by our advertising partners. They may be used by those companies to build a profile of your interests and show you relevant adverts on other sites. If you do not allow these cookies, you will experience less targeted advertising.

Cookie List

Clear

Apply Cancel

Consent Leg.Interest

Confirm my choices

Powered by onetrust
