# Supplementary material for: Spatial integration of multi-omics data from serial sections using the novel Multi-Omics Imaging Integration Toolset
Source: Gigascience. 2025 May 14;14:giaf035. doi: 10.1093/gigascience/giaf035 (PMC12077394; doi:10.1093/gigascience/giaf035)
Supplement: giaf035_Reviewer_1_Original_Submission — Hua Zhang -- 11/20/2024 [file giaf035_reviewer_1_original_submission.pdf]

Reviewer Report

Title: Spatial Integration of Multi-Omics Data from Serial Sections using the novel Multi-Omics Imaging Integration Toolset

Version: Original Submission **Date:** 11/20/2024

Reviewer name: Hua Zhang

Reviewer Comments to Author:

Wess et al reports a Python framework, MIIT (Multi-Omics Imaging Integration Toolset), for integrating spatially resolved multi-omics data. Multi-omics imaging represents a pivotal approach for systems molecular biology and biomarker discovery. This method introduces a timely and valuable tool to advance the field. However, in my opinion, this paper still has some issues that need to be addressed before consideration for publication.Cancer tissue exhibits significant heterogeneity effects, in this study, different molecular information obtaining from different tissue sections, this means from different cells as the tissue section is 10 um thickness, almost the diameter of the cells. Please height the meaningful of co-registration information if they are obtained from different cell layers. In particular, for the datasets of spatial transcriptomics and MSI, the experiments were conducted on serial sections with an axial sectioning distance of 40 to 100 Î¼m. This means that the mRNA and metabolites originate from different cells, raising questions about how integrating these two datasets can provide meaningful insights.The multi-omics imaging integration toolset is based on the GreedyFHist, a non-rigid registration algorithm, it suggests including more details about this algorithm and highlight the difference comparing to previously reported non-rigid image co-registration algorithm.The author should demonstrate the accuracy of background segmentation, it concerns certain low signal sample area would be removed in the denoising step. What is criterion to define the background region and sample region in the background segmentation.In the Method section, more details need to be included in the spatial transcriptomics part, what the spatial resolution of the 10x Genomics was used. As the MALDI resolution is 30 um, how the pixel alignment of the ST and MSI data if their spatial resolution is different.In the MALDI-MSI of prostate tissue, on tissue MS/MS data is missing to confirm the identification of target analytes of citrate, $ZnCl_3^-$, and spermine.

Level of Interest

Please indicate how interesting you found the manuscript: Choose an item.

Quality of Written English

Please indicate the quality of language in the manuscript: Choose an item.

Declaration of Competing Interests

Please complete a declaration of competing interests, considering the following questions:

- Have you in the past five years received reimbursements, fees, funding, or salary from an organisation that may in any way gain or lose financially from the publication of this manuscript, either now or in the future?
- Do you hold any stocks or shares in an organisation that may in any way gain or lose financially from the publication of this manuscript, either now or in the future?
- Do you hold or are you currently applying for any patents relating to the content of the manuscript?
- Have you received reimbursements, fees, funding, or salary from an organization that holds or has applied for patents relating to the content of the manuscript?
- Do you have any other financial competing interests?
- Do you have any non-financial competing interests in relation to this paper?

If you can answer no to all of the above, write 'I declare that I have no competing interests' below. If your reply is yes to any, please give details below.

I declare that I have no competing interests

I agree to the open peer review policy of the journal. I understand that my name will be included on my report to the authors and, if the manuscript is accepted for publication, my named report including any attachments I upload will be posted on the website along with the authors' responses. I agree for my report to be made available under an Open Access Creative Commons CC-BY license (http://creativecommons.org/licenses/by/4.0/). I understand that any comments which I do not wish to be included in my named report can be included as confidential comments to the editors, which will not be published.

Choose an item.

To further support our reviewers, we have joined with Publons, where you can gain additional credit to further highlight your hard work (see: https://publons.com/journal/530/gigascience). On publication of this paper, your review will be automatically added to Publons, you can then choose whether or not to claim your Publons credit. I understand this statement.

Yes Choose an item.
