# Supplementary material for: Spatial integration of multi-omics data from serial sections using the novel Multi-Omics Imaging Integration Toolset
Source: Gigascience. 2025 May 14;14:giaf035. doi: 10.1093/gigascience/giaf035 (PMC12077394; doi:10.1093/gigascience/giaf035)
Supplement: giaf035_Reviewer_2_Original_Submission — Santhoshi Krishnan -- 11/26/2024 [file giaf035_reviewer_2_original_submission.pdf]

Reviewer Report

Title: Spatial Integration of Multi-Omics Data from Serial Sections using the novel Multi-Omics Imaging Integration Toolset

Version: Original Submission **Date:** 11/26/2024

Reviewer name: Santhoshi Krishnan

Reviewer Comments to Author:

Overview:In this paper, the authors present the Multi-Omics Imaging Integration Toolset, which is a python framework for integration multiple spatial omics datatypes. To facilitate this, they also development a registration method (GreedyFHist) for jointly analyzing sequential tissue layers that have undergone different types of staining/phenotyping regimens. The method validation was done on a 244 fresh-frozen prostrate tissue sections. The highly detailed methods and results section is well appreciated and helps fully contextualize the significance of the study. The definitions of study-specific terms mentioned throughout the paper at the beginning are also appreciated.Data and Code Availability:Detailed code, tutorials and associated instructions have been made available for use by the public, which is appreciated. All systems requirements have also been explicitly laid out for ease of installation and use. The workflow examples provided are quite detailed; however, a more extensive codebase with stepwise explanations within the code will be appreciated.Data has not been made available publicly, except for the raw and processed spatial transcriptomics data; however, detailed and explicit instructions have been provided on data access, keeping in mind local regulations.Revisions:Major Revisions:1. In recent years, a lot of other platforms, both free and paid, tend to support registration across multiple slides. For example, HALO has a registration feature available as well, along with a host of other open-source datatypes. In that regard, how is your platform different?2. It is mentioned that downscaling occurs during the registration process in order to reduce runtime - how are nuances in features selected as registration landmarks preserved in such a case?3. How is the fixed image determined in this case? The assumption would be that a standard H&E image is selected for this purpose- is that assumption, correct?4. The authors have stated and justified their rationale for using the mentioned evaluation metrics in the paper. However, in the general image registration space, metrics such as the dice coefficient and jaccard index are commonly used and accepted. Is there a particular reason why these were not used as well? It would offer a more complete picture for the general user if these metrics were provided as well.5. The validation of registering distance neighboring sections is quite a valuable contribution, as the authors rightly stated that in many multi-omics experiments, this might be a necessity. However, when looking at tissue sections that are 80-100 microns apart, it is quite likely that the set of cells that one may be looking at on the x-y coordinate system may not be the same at all; in fact, for a highly heterogeneous/flexible piece of tissue, they might be completely different. In such a circumstance, how much value is there in registering these two sections together instead of, say, separately analyzing them and using alternative methods to combine the results downstream?6. In the proof of concept presented in the paper, the authors mention using ST and MSI data for validating their framework. Have they also investigated ST

integration with more commonly available datatypes such as IHC/mIF?7. The work that the authors have put in to validate the registration and MIIT framework using different approaches (selecting spatially distant slides, integration using augmented/artificial data) is thorough. However, different tissue types bring in their own challenges, and thus validation of this framework on an external dataset would lend more credence to this much needed framework, especially in the era of increased multiomics analyses.Minor Revisions:1. Please ensure all typos/grammatical mistakes are corrected.2. In the 'preprocessing of stained histology images', can more details be given on the thresholding process? It is also stated that the threshold is manually adjusted for each image if necessary - how is this determination done?3. The headings/subheadings organizations within sections can be done in a more organized manner, in some parts it was challenging to determine the organization of sections/subsections.4. Can some more details be given on the landmarks that were identified per image? Could some examples be provided on what these landmarks are, and how they remain consistence across tissue layers?5. Currently, the way various samples are used for validating the GreedyFHist and MIIT frameworks are listed out in the paper is quite confusing. It would be appreciated if the authors can distinctly mention the number of samples out of the set of samples, and the associated stained slides are used for each.6. How were the annotations from the 3 annotators cross-validated?

Level of Interest

Please indicate how interesting you found the manuscript: Choose an item.

Quality of Written English

Please indicate the quality of language in the manuscript: Choose an item.

Declaration of Competing Interests

Please complete a declaration of competing interests, considering the following questions:

- Have you in the past five years received reimbursements, fees, funding, or salary from an organisation that may in any way gain or lose financially from the publication of this manuscript, either now or in the future?
- Do you hold any stocks or shares in an organisation that may in any way gain or lose financially from the publication of this manuscript, either now or in the future?
- Do you hold or are you currently applying for any patents relating to the content of the manuscript?
- Have you received reimbursements, fees, funding, or salary from an organization that holds or has applied for patents relating to the content of the manuscript?
- Do you have any other financial competing interests?
- Do you have any non-financial competing interests in relation to this paper?

If you can answer no to all of the above, write 'I declare that I have no competing interests' below. If your reply is yes to any, please give details below.

I declare that I have no competing interests.

I agree to the open peer review policy of the journal. I understand that my name will be included on my report to the authors and, if the manuscript is accepted for publication, my named report including any attachments I upload will be posted on the website along with the authors' responses. I agree for my report to be made available under an Open Access Creative Commons CC-BY license (http://creativecommons.org/licenses/by/4.0/). I understand that any comments which I do not wish to be included in my named report can be included as confidential comments to the editors, which will not be published.

Choose an item.

To further support our reviewers, we have joined with Publons, where you can gain additional credit to further highlight your hard work (see: https://publons.com/journal/530/gigascience). On publication of this paper, your review will be automatically added to Publons, you can then choose whether or not to claim your Publons credit. I understand this statement.

Yes Choose an item.
